# Synthesis and structure of 4-hy­droxy-*N*-iso­propyl­tryptamine (4-HO-NiPT) and its precursors

**DOI:** 10.1107/S2056989023002098

**Published:** 2023-03-10

**Authors:** Uroš Laban, Marilyn Naeem, Andrew R. Chadeayne, James A. Golen, David R. Manke

**Affiliations:** a CaaMTech, Inc., 58 East Sunset Way, Suite 209, Issaquah, WA 98027, USA; b University of Massachusetts Dartmouth, 285 Old Westport Road, North Dartmouth, MA 02747, USA; University of Aberdeen, United Kingdom

**Keywords:** crystal structure, tryptamines, indoles, hydrogen bonding

## Abstract

The title compound, 4-hy­droxy-*N*-iso­propyl­tryptamine (**4**) or 4-HO-NiPT (systematic name: 3-{2-[(propan-2-yl)amino]­eth­yl}-1*H*-indol-4-ol), C_13_H_18_N_2_O, was synthesized in three steps from 4-benzyl­oxyindole (**1**) (systematic name: 4-phen­oxy-1*H*-indole), C_15_H_13_NO. (**1**) was treated with oxalyl chloride and iso­propyl­amine to produce *N*-isopropyl-4-benz­yloxy-3-indole­glyoxyl­amide (**2**) {systematic name: 2-[4-(benz­yloxy)-1*H*-indol-3-yl]-2-oxo-*N*-(propan-2-yl)acet­amide}, C_20_H_20_N_2_O_3_. (**2**) was reduced to generate 4-benz­yloxy-*N*-iso­propyl­tryptamine **(3)** or 4-HO-NiPT, which was characterized as its chloride salt 4-benz­yloxy-*N*-iso­propyl­tryptammonium chloride (**3a**) (systematic name: {2-[4-(benz­yloxy)-1*H*-indol-3-yl]eth­yl}(propan-2-yl)aza­nium chloride), C_20_H_25_N_2_O·Cl. Finally the benzyl group of **(3)** was removed *via* hydrogenation to generate 4-HO-NiPT. The crystal structures of the title compound and all three synthetic precursors are presented.

## Abstract 2

The synthesis of the norpsilocin derivative, 4-hy­droxy-*N*-iso­propyl­tryptamine, is presented, as well as its crystal structure and the structures of its three synthetic precursors.

## 1. Chemical context

Psilocybin (C_12_H_17_N_2_O_4_P, 4-phosphor­yloxy-*N*,*N*-di­methyl­tryptamine) has recently garnered a great deal of inter­est due to its potential to ameliorate a number of treatment resistant mood disorders (Carhart-Harris & Goodwin, 2017 ▸; Nichols *et al.*, 2017 ▸). Upon administration, psilocybin is enzymatically metabolized *via* hydrolysis of its 4-phosphor­yloxy group, producing psilocin (C_12_H_16_N_2_O, 4-hy­droxy-*N*,*N*-di­methyl­tryptamine) as the active metabolite. Psilocin is an agonist of the serotonin 2A (5-HT_2A_) receptor; this activity is believed to be responsible for producing a head-twitch response (HTR) in murine models, as well as subjective ‘psychedelic’ effects in human subjects as well as other potentially beneficial biological and clinical results (Halberstadt *et al.*, 2020 ▸).

Psilocybin and psilocin are both natural products, found in over 200 species of ‘magic mushrooms’ (Stamets, 1996 ▸). However, psilocybin and psilocin are not the only tryptamines present in these fungi. Other structurally similar mol­ecules have been observed in significant qu­anti­ties. (Leung & Paul, 1968 ▸; Jensen *et al.*, 2006 ▸; Lenz *et al.*, 2017 ▸). Such structural analogs include baeocystin (C_11_H_15_N_2_O_4_P, 4-phosphor­yloxy-*N*-methyl­tryptamine), the monomethyl derivative of psilocybin, and aerugeniscin (C_13_H_20_N_2_O_4_P, 4-phosphor­yloxy-*N*,*N,N*-tri­methyl­tryptamine), its trimethyl variant. Like psilocybin, both baeocystin and aeruginascin are 4-phospho­ryloxytryptamines, which are hydro­lized to their analogous 4-hy­droxy­tryptamines: norpsilocin (C_11_H_14_N_2_O) and 4-hy­droxy-*N*,*N*,*N*-tri­methyl­tryptamine respectively.

In the case of baeocystin, its active metabolite norpsilocin (4-hy­droxy-*N*-methyl­tryptamine) has been examined and shown to be a full agonist of the 5-HT_2A_ receptor (Sherwood *et al.*, 2020 ▸; Glatfelter *et al.*, 2022*b* ▸). Notably, despite this activity, norpsilocin does not show a head-twitch response (HTR) in mice, the standard animal test to indicate a psychedelic-like response. Unlike dialkyl tryptamines (*e.g*., psilocybin and psilocin) the pharmacology of analogous monoalkyl tryptamines (*e.g*., baeocystin and norpsilocin) is relatively unknown. Accordingly, the importance of these compounds within the context of the overall polypharmacological ‘magic mushroom’ experience is not understood. In an effort to explore the proprieties of mono­alkyl­tryptamines, we previously reported the structural characterization, cellular, and behavioral data for baeocystin and norpsilocin (Naeem *et al.*, 2022 ▸; Chadeayne *et al.*, 2020*b* ▸). 
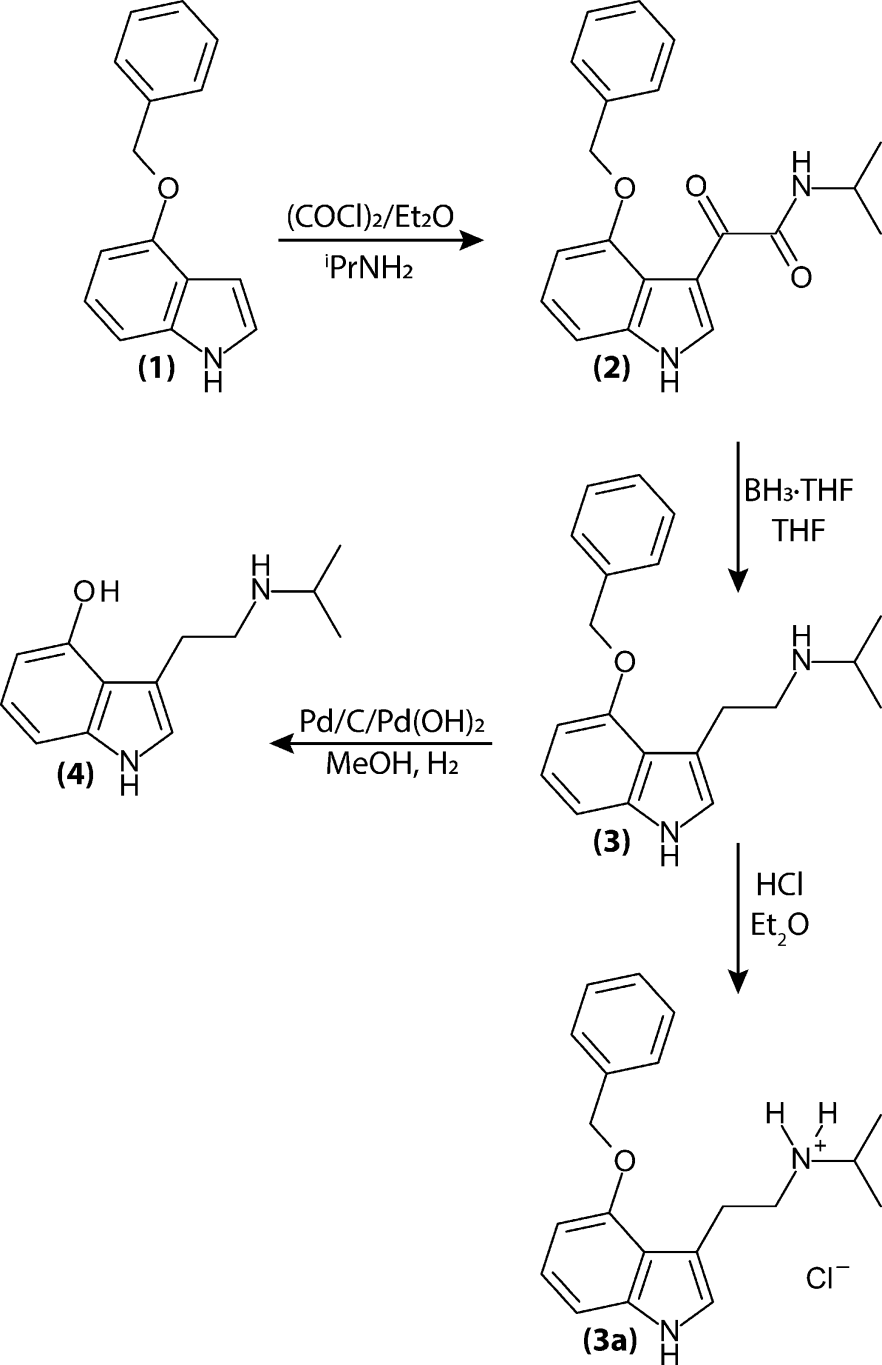


Herein, we expand our exploration to include other 4-hy­droxy monoalkyl tryptamines in an effort to examine how the steric variation impacts serotonergic activity. Our first target, 4-hy­droxy-*N*-iso­propyl­tryptamine (4-HO-NiPT), replaces the methyl group of norpsilocin with an isopropyl group. The only previous literature report of this mol­ecule is as a metabolite of the new psychoactive substance (NPS) 4-acet­oxy-*N*,*N*-diisoproptyltryptamine (4-AcO-DiPT) from 2022 (Malaca *et al.*, 2022 ▸). The synthesis of the title compound, 4-HO-NiPT, follows a procedure modified from the psilocin synthesis put forward by Albert Hofmann in 1959 (Troxler *et al.*, 1959 ▸). The structure of 4-HO-NiPT and those of its three synthetic precursors are reported herein.

## 2. Structural commentary

**Figure 1:**
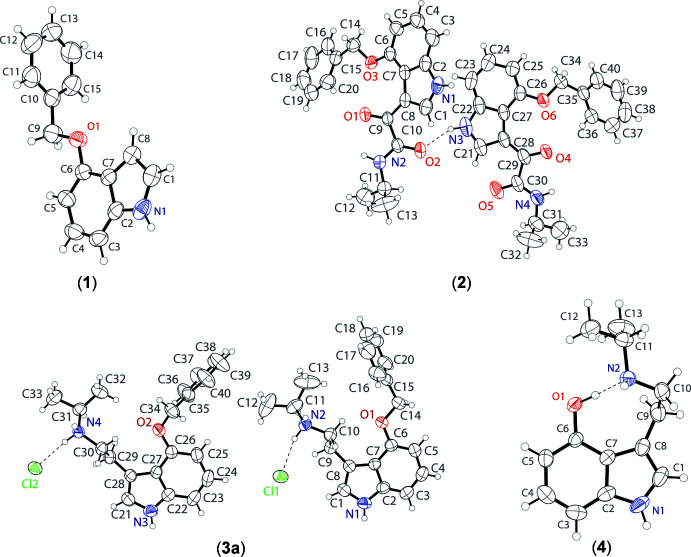
The mol­ecular structures of 4-benzyl­oxyindole (**1**) (top left), *N*-2-propyl-α-oxo-4-(phenyl­meth­oxy)-1*H*-indole-3-acetamide (**2**) (top right), 4-benz­yloxy-*N*-iso­propyl­ammonium chloride (**3a**) (bottom left), and 4-hy­droxy-*N*-iso­propyl­tryptamine (**4**) (bottom right), showing the atomic labeling. Displacement ellipsoids are drawn at the 50% probability level. Hydrogen bonds are shown as dashed lines.

The asymmetric unit of 4-benzyl­oxyindole, C_15_H_13_NO (**1**) contains a single mol­ecule (Fig. 1 ▸). The indole ring system of the tryptamine grouping is almost planar with an r.m.s. deviation from planarity of 0.013 Å. The benz­yloxy group has an *anti* conformation with a C6—O1—C9—C10 torsion angle of −179.00 (13)°. The benzene ring of the benz­yloxy group is near perpendicular from the indole ring with a plane to plane twist of 89.73 (6)°.

The asymmetric unit of *N*-2-propyl-α-oxo-4-(phenyl­meth­oxy)-1*H*-indole-3-acetamide, C_20_H_20_N_2_O_3_ (**2**) contains two mol­ecules of the indole-amide (Fig. 1 ▸). The indole rings of both mol­ecules are near planar, with r.m.s. deviations from planarity of 0.021 Å and 0.011 Å for the N1 and N3 mol­ecules, respectively. The benz­yloxy groups both have *anti* conformations with a C6—O3—C14—C15 torsion angle of 179.6 (4)° and a C26—O6—C34—C35 angle of −178.2 (4)°. The benzene rings of the benz­yloxy groups are slightly twisted from the indole rings with plane-to-plane (dihedral) twists of 19.98 (16) and 21.45 (16)°, respectively. The amide arms are slightly turned away from the indole rings with a C7—C8—C9—C10 angle of 157.3 (4)° and a C27—C28—C29—C30 angle of −160.8 (4)°. The amine groups are in *anti* conformations with C8—C9—C10—N2 = 165.2 (4)° and C28—C29—C30—N4 = 174.3 (4)°. The isopropyl groups are also in *anti* conformations with a C9—C10—N2—C11 angle of −178.1 (4)° and a C29—C30—N4—C31 angle of 178.5 (4)°. In the asymmetric unit, the indole–amide mol­ecules are linked by an N—H⋯O hydrogen bond between the indole nitro­gen and the carbonyl oxygen of the amide group.

The asymmetric unit of 4-benz­yloxy-*N*-iso­propyl­tryptammonium chloride, C_20_H_25_N_2_O^+^·Cl^−^ (**3a**) contains two tryptammonium cations (identified by atoms N1 and N3) and two chloride anions (Fig. 1 ▸). The indole rings of both cations are close to planar, with r.m.s. deviations of 0.004 Å and 0.010 Å for the N1 and N3 cations, respectively. The benz­yloxy group of the N1 cation shows an *anti* conformation with a C6—O1—C14—C15 angle of −173.52 (17)°; in the N3 cation, the benz­yloxy group is turned nearly perpendicular to the indole ring with a C26—O2—C34—C35 angle of 85.8 (2)°. In both cations, the ethyl­amino arms are turned away from the indole rings, with a C7—C8—C9—C10 angle of 73.5 (2)° and a C27—C28—C29—C30 angle of 72.3 (2)°. The amine groups of the arm are both in *anti* conformations with C8—C9—C10—N2 = −153.55 (16)° and C28—C29—C30—N4 = −172.94 (14)°. On the contrary, the isopropyl groups are in *syn* conformations with a C9—C10—N2—C11 angle of −59.7 (2)° and a C29—C30—N4—C31 angle of −54.9 (2)°. In the asymmetric unit, the tryptammonium cations and chloride anions are linked by N—H⋯Cl hydrogen bonds arising from the indole nitro­gen atoms.

The asymmetric unit of 4-hy­droxy-*N*-iso­propyl­tryptamine C_13_H_18_N_2_O (**4**) contains a single tryptamine mol­ecule (Fig. 1 ▸). The indole ring system is almost planar with an r.m.s. deviation of 0.006 Å. The ethyl­amine arm of the tryptamine is turned away from the indole ring, with a C7—C8—C9—C10 torsion angle of 75.2 (2)° whereas the C8—C9—C10—N2 torsion angle of −72.6 (2)° turns the amine group back toward the hydroxide substituent on the 4-position of the indole ring system. The turn of the ethyl­amine arm is due to an intra­molecular O—H⋯N hydrogen bond between the hydroxide group and the amine N atom.

## 3. Supra­molecular features

**Figure 2:**
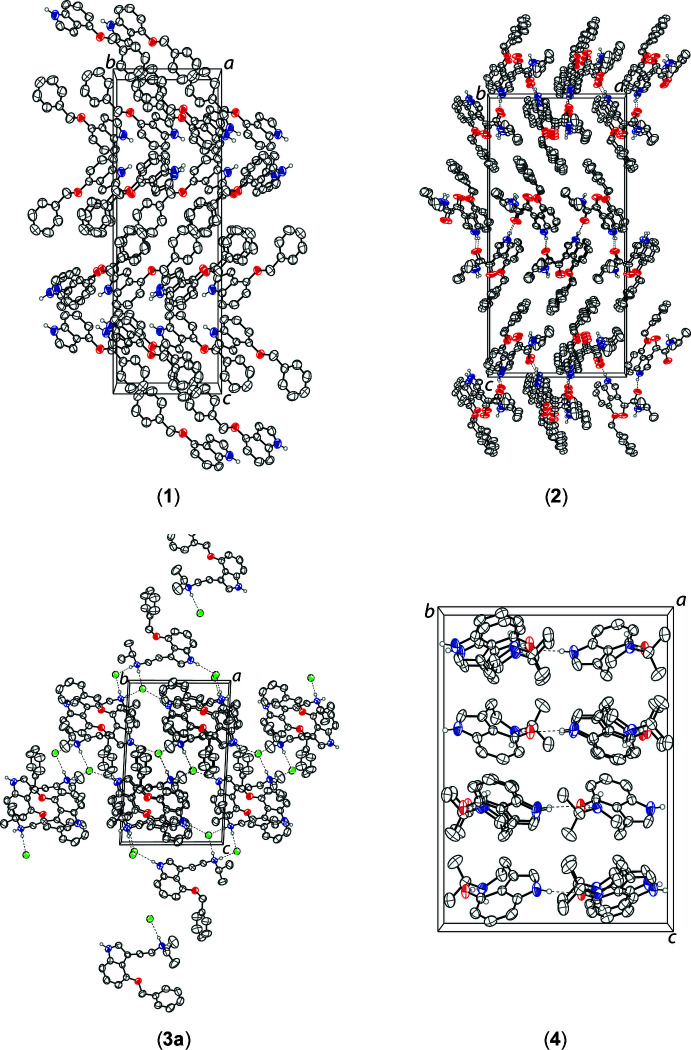
The crystal packing of 4-benzyl­oxyindole (**1**) along the *a* axis (top left), *N*-2-propyl-α-oxo-4-(phenyl­meth­oxy)-1*H*-indole-3-acetamide (**2**) along the *b* axis (top right), 4-benz­yloxy-*N*-iso­propyl­ammonium chloride (**3a**) along the *a* axis (bottom left), and 4-hy­droxy-*N*-iso­propyl­tryptamine (**4**) along the *a* axis (bottom right). The hydrogen bonds (Tables 2 ▸–4 ▸ ▸) are shown as dashed lines. Hydrogen atoms not involved in hydrogen bonds are omitted for clarity.

There are no significant inter­molecular inter­actions in (**1**) beyond normal van der Waals contacts. The mol­ecules of (**2**) are linked by N—H⋯O hydrogen bonds, generating infinite chains along the [100] direction between indole N atoms and the O atoms of the amide carbonyl groups (Table 1 ▸). The tryptammonium cations and chloride anions of (**3a**) are linked into infinite chains propagating along [010] by N—H⋯Cl hydrogen bonds between the indole nitro­gen atoms and the chloride anions and the ammonium N atoms and the Cl^−^ ions (Table 2 ▸). The tryptamine mol­ecules of (**4**) are held together in infinite chains along the [010] direction by N—H⋯O hydrogen bonds between the indole NH groupings and the hydroxide O atoms (Table 3 ▸). The crystal packing of compounds (**1**)–(**4**) are shown in Fig. 2 ▸.

## 4. Database survey

The most closely related structure to the title compound is norpsilocin, 4-hy­droxy-*N*-methyl­tryptamine, which has been reported as its free base and its fumarate salt [Cambridge Structural Database (Groom *et al.*, 2016 ▸) refcodes MULXAV and MULXEZ: Chadeayne *et al.*, 2020*a* ▸]. Norpsilocin free base has its ethyl­amino arm in an *anti* conformation, with O—H⋯N hydrogen bonds being inter­molecular rather than intra­molecular like compound (**4**). There are three other mono­alkyl­tryptamines reported in the literature, the natural product baeocystin (FEJBAB: Naeem *et al.*, 2022 ▸), 4-acet­oxy-*N*-methyl­tryptamine (Glatfelter *et al.*, 2022*b* ▸) and 5-meth­oxy-*N*-methyl­tryptamine (QQQAHA: Bergin *et al.*, 1968 ▸). There are nine other 4-hy­droxy­tryptamines reported in the literature, the natural product psilocin (PSILIN: Petcher & Weber, 1974 ▸), 4-hy­droxy-*N*-methyl-*N*-iso­propyl­tryptamine as its fumarate (TUFQAP: Chadeayne *et al.*, 2020*a* ▸) and hydro­fumarate (RONSUL: Chadeayne *et al.*, 2019*a* ▸) salts, 4-hy­droxy-*N*,*N*-di-*n*-propyl­tryptamine as its chloride (WAMGEA: Sammeta *et al.*, 2020 ▸) and fumarate (WUCGAF: Chadeayne *et al.*, 2019*b* ▸) salts, and the four quaternary tryptamines 4-hy­droxy-*N*,*N*,*N*-tri­methyl­tryptamine (XUXFAA: Chadeayne *et al.*, 2020*c* ▸), 4-hy­droxy-*N*,*N*-dimethyl-*N*-ethyl­tryptamine, 4-hy­droxy-*N*,*N*-di­methyl­iso­propyl­tryptamine, and 4-hy­droxy-*N*,*N*-di-*n*-propyl­tryptamine (EDOYIJ, EDOYUV and EDOZIK: Glatfelter *et al.*, 2022*a* ▸). The reported structures most closely related to compound (**2**) are indole-3-yl-*N*-iso­propyl­glyoxalyl­amide (HUNCID: Mansell *et al.*, 2009 ▸), 4-benzyl­oxyindole-3-yl-*N*,*N*-diiso­propyl­glyoxalyl­amide (RUHYEY: Spaeth *et al.*, 1997 ▸) and 4-acet­oxy­indole-3-yl-*N*,*N*-di­ethyl­glyoxalyl­amide (AVUMOT: Wu *et al.*, 2004 ▸).

## 5. Synthesis and crystallization

**4-Benzyl­oxyindole (1):** Single crystals of (**1**) were grown from the vapor diffusion of diethyl ether into a methyl­ene chloride solution of a commercial sample (Biosynth).

***N*-isopropyl-4-benz­yloxy-3-indole­glyoxyl­amide **(2):**** to a solution of (**1**) (2.0 g, 8.96 mmol) in di­ethyl­ether (50 ml) was added oxalylchloride (2.3 g, 17.93 mmol) dropwise at 273 K. The resulting mixture was stirred for 6 h at 273 K, and 2-propyl­amine (4.24 g, 71.68 mmol) was added dropwise. The mixture was warmed to room temperature and stirred overnight. Solvent was removed *in vacuo*, and the resulting residue was purified on a silica gel column (methyl­ene chloride/methanol) to afford the product as a yellow oil (2.9 g, 96% yield). Single crystals of (**2**) suitable for X-ray diffraction studies were grown by vapor diffusion of diethyl ether into a methyl­ene chloride solution.

**4-Benz­yloxy-** ***N*** **-iso­propyl­tryptamine (3):** To a suspension of (**2**) (900 mg, 2.68 mmol) in tetra­hydro­furan (12 ml) was added borane–tetra­hydro­furan complex (1.0 *M*, 8.0 ml, 8.0 mmol) dropwise at 273 K. The reaction was then heated at reflux overnight. The resulting yellow solution was cooled to room temperature and quenched with hydro­chloric acid (2.0 *M*), then heated at 273 K for 2 h. The mixture was cooled to room temperature and ammonium hydroxide was added until the pH exceeded 8. The mixture was extracted with methyl­ene chloride and the organic layer was washed with water and brine, dried over sodium sulfate, and solvent was removed *in vacuo*. The residue was purified by silica gel column chromatography (methyl­ene chloride/ammonia-methanol solution) to afford **(3)** as a yellow solid (190 mg, 23% yield). The compound was treated with hydro­chloric acid (1.0 *M* in di­ethyl­ether) and filtered to yield the chloride salt (**3a**). Single crystals of (**3a**) suitable for X-ray diffraction studies were grown from the vapour diffusion of di­ethyl­ether into a methanol solution.^1^H NMR (400 MHz, CDCl_3_): δ 8.11 (*br s*, 1H, N*H*), 7.49 (*d*, *J* = 7.2 Hz, 2H, Ar*H*), 7.41 (*t*, *J* = 7.2 Hz, 2H, Ar*H*), 7.35 (*d*, *J* = 7.2 Hz, 1H, Ar*H*), 7.08 (t, *J* = 8.0 Hz, 1H, Ar*H*), 6.99 (d, *J* = 8.0 Hz, 1H, Ar*H*), 6.96 (*s*, 1H, Ar*H*), 6.57 (*d*, *J* = 8.0 Hz, 1H, Ar*H*), 3.11 (*t*, *J* = 7.2 Hz, 2H, C*H*_2_), 2.93 (*t*, *J* = 7.2 Hz, 2H, C*H*_2_), 2.77–2.71 (*m*, 1H, C*H*), 0.99 (*d*, *J* = 6.4 Hz, 6H, C*H*_3_).

**4-Hy­droxy-** ***N*** **-iso­propyl­tryptamine (4):** To a solution of **(3)** (190 mg, 0.62 mmol) in methanol (4.0 ml) was added palladium on carbon (30 mg) and palladium hydroxide on carbon (30 mg). The mixture was stirred for 3 h under an atmosphere of hydrogen. The resulting black suspension was filtered and washed with methanol. Solvent was removed *in vacuo* and the resulting residue was purified by silica gel chromatography (methyl­ene chloride/ammonia methanol solution) to afford (**4**) as an off-white solid (87 mg, 64% yield). Single crystals of (**4**) suitable for X-ray diffraction studies were grown from the slow evaporation of an acetone solution. ^1^H NMR (400 MHz, CDCl_3_): δ 7.88 (*s*, 1H, N*H*), 7.05 (*t*, *J* = 7.9 Hz, 1H, Ar*H*), 6.90–6.79 (*m*, 2H, Ar*H*), 6.59 (*d*, 1H, Ar*H*), 3.02 (*t*, 2H, C*H*_2_), 2.96 (*t*, 2H, C*H*_2_), 2.91–2.83 (*m*, 1H, C*H*), 1.12 (*d*, *J* = 6.4 Hz, 6H, C*H*_3_). ^13^C NMR (101 MHz, CDCl_3_): δ 151.9 (Ar*C*), 138.7 (Ar*C*), 123.5 (Ar*C*), 120.8 (Ar*C*), 118.5 (Ar*C*), 114.0 (Ar*C*), 107.0 (Ar*C*), 102.7 (Ar*C*), 49.5 (Ak*C*), 48.0 (Ak*C*), 27.6 (Ak*C*), 22.1 (Ak*C*). MS (ESI) calculated for C_13_H_16_N_2_O: 218.1; found: 218.9 [*M* + 1].

## 6. Refinement

Crystal data, data collection and structure refinement details are summarized in Table 4 ▸. Hydrogen atoms H1 in compound (**1**), H1, H2, H3*A* and H4*A* in compound (**2**), H1, H2*A*, H2*B*, H3*A*, H4*A* and H4*B* in compound (**3a**) and H1, H1*A* and H2 in compound (**4**) were found from difference-Fourier maps and were refined isotropically. DFIX restraints were used on all of these hydrogen atoms [except H1 in compound (**1**), which was refined freely] with N—H distances of 0.87 (1) Å for the indole N atoms, 0.90 (1) Å for the ethyl­amino N atoms, 0.95 (1) Å for ethyl­ammonium N atoms, and 1.00 (1) Å for the O–H distance. Isotropic displacement parameters were set to 1.2 *U*_eq_ of the indole N atoms and 1.5 *U*_eq_ of the parent ethyl­amino N atoms and the parent oxygen atom. All other hydrogen atoms were placed in calculated positions (C—H = 0.93–0.98 Å). Isotropic displacement parameters were set to 1.2 *U*_eq_ (C) or 1.5 *U*_eq_ (C-meth­yl).

## Supplementary Material

Crystal structure: contains datablock(s) 1, 2, 3a, 4, global. DOI: 10.1107/S2056989023002098/hb8057sup1.cif

Structure factors: contains datablock(s) 1. DOI: 10.1107/S2056989023002098/hb80571sup2.hkl

Structure factors: contains datablock(s) 2. DOI: 10.1107/S2056989023002098/hb80572sup3.hkl

Structure factors: contains datablock(s) 3a. DOI: 10.1107/S2056989023002098/hb80573asup4.hkl

Structure factors: contains datablock(s) 4. DOI: 10.1107/S2056989023002098/hb80574sup5.hkl

Supporting information file. DOI: 10.1107/S2056989023002098/hb80571sup6.cml

Supporting information file. DOI: 10.1107/S2056989023002098/hb80572sup7.cml

Supporting information file. DOI: 10.1107/S2056989023002098/hb80573sup8.cml

Supporting information file. DOI: 10.1107/S2056989023002098/hb80574sup9.cml

CCDC references: 2246617, 2246618, 2246619, 2246620

Additional supporting information:  crystallographic information; 3D view; checkCIF report

## Figures and Tables

**Table 1:** Hydrogen-bond geometry (Å, °) for (**2**)

*D*—H⋯*A*	*D*—H	H⋯*A*	*D*⋯*A*	*D*—H⋯*A*
N1—H1⋯O5^i^	0.86	1.98	2.810 (5)	164
N3—H3*A*⋯O2	0.87 (2)	1.99 (3)	2.801 (5)	154 (5)

Symmetry code: (i) 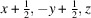 .

**Table 2:** Hydrogen-bond geometry (Å, °) for (**3a**)

*D*—H⋯*A*	*D*—H	H⋯*A*	*D*⋯*A*	*D*—H⋯*A*
N1—H1⋯Cl1^i^	0.87 (1)	2.36 (1)	3.1969 (17)	162 (2)
N2—H2*A*⋯Cl1^ii^	0.94 (1)	2.18 (1)	3.1114 (16)	167 (2)
N2—H2*B*⋯Cl1	0.95 (1)	2.23 (1)	3.1191 (16)	157 (2)
N3—H3*A*⋯Cl2^iii^	0.86 (1)	2.42 (1)	3.2657 (17)	168 (2)
N4—H4*A*⋯Cl2^iv^	0.94 (1)	2.20 (1)	3.1360 (16)	173 (2)
N4—H4*B*⋯Cl2	0.94 (1)	2.19 (1)	3.1247 (16)	171 (2)

Symmetry codes: (i) 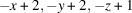 ; (ii) 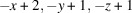 ; (iii) 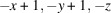 ; (iv) 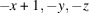 .

**Table 3:** Hydrogen-bond geometry (Å, °) for (**4**)

*D*—H⋯*A*	*D*—H	H⋯*A*	*D*⋯*A*	*D*—H⋯*A*
N1—H1*A*⋯O1^i^	0.88 (1)	2.06 (1)	2.9217 (16)	167 (2)
O1—H1⋯N2	1.00 (1)	1.62 (1)	2.6217 (15)	176 (2)

Symmetry code: (i) 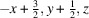 .

**Table 4:** Experimental details

	(**1**)	(**2**)	(**3a**)	(**4**)
Crystal data				
Chemical formula	C_15_H_13_NO	C_20_H_20_N_2_O_3_	C_20_H_25_N_2_O^+^·Cl^−^	C_13_H_18_N_2_O
*M*_r_	223.26	336.38	344.87	218.29
Crystal system, space group	Orthorhombic, *P* *b* *c* *a*	Orthorhombic, *P* *n* *a*2_1_	Triclinic, *P* 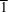	Orthorhombic, *P* *b* *c* *a*
Temperature (K)	300	300	300	300
*a*, *b*, *c* (Å)	9.8201 (9), 9.0067 (7), 26.995 (2)	16.4230 (13), 6.5609 (4), 33.469 (2)	10.1895 (9), 10.9117 (7), 17.6887 (14)	8.4065 (5), 14.3944 (9), 19.8501 (10)
α, β, γ (°)	90, 90, 90	90, 90, 90	86.798 (3), 79.340 (3), 87.587 (3)	90, 90, 90
*V* (Å^3^)	2387.6 (4)	3606.2 (4)	1928.7 (3)	2402.0 (2)
*Z*	8	8	4	8
Radiation type	Mo *K*α	Mo *K*α	Mo *K*α	Mo *K*α
μ (mm^−1^)	0.08	0.08	0.21	0.08
Crystal size (mm)	0.40 × 0.22 × 0.12	0.34 × 0.30 × 0.08	0.30 × 0.27 × 0.20	0.33 × 0.25 × 0.20

Data collection				
Diffractometer	Bruker D8 Venture CMOS	Bruker D8 Venture CMOS	Bruker D8 Venture CMOS	Bruker D8 Venture CMOS
Absorption correction	Multi-scan (*SADABS*; Bruker, 2021 ▸)	Multi-scan (*SADABS*; Bruker, 2021 ▸)	Multi-scan (*SADABS*; Bruker, 2021 ▸)	Multi-scan (*SADABS*; Bruker, 2021 ▸)
*T*_min_, *T*_max_	0.697, 0.745	0.694, 0.745	0.658, 0.745	0.715, 0.745
No. of measured, independent and observed [*I* > 2σ(*I*)] reflections	56335, 2451, 2098	48212, 6584, 5295	42151, 7362, 5589	61022, 2461, 2121
*R*_int_	0.039	0.037	0.033	0.050
(sin θ/λ)_max_ (Å^−1^)	0.626	0.602	0.612	0.626

Refinement				
*R*[*F* ^2^ > 2σ(*F* ^2^)], *wR*(*F* ^2^), *S*	0.045, 0.110, 1.11	0.046, 0.117, 1.04	0.041, 0.113, 1.02	0.045, 0.116, 1.08
No. of reflections	2451	6584	7362	2461
No. of parameters	159	468	461	159
No. of restraints	0	4	6	3
H-atom treatment	H atoms treated by a mixture of independent and constrained refinement	H atoms treated by a mixture of independent and constrained refinement	H atoms treated by a mixture of independent and constrained refinement	H atoms treated by a mixture of independent and constrained refinement
Δρ_max_, Δρ_min_ (e Å^−3^)	0.16, −0.12	0.17, −0.15	0.26, −0.29	0.18, −0.15
Absolute structure	–	Refined as an inversion twin	–	–
Absolute structure parameter	–	0.1 (18)	–	–

Computer programs: *APEX4* (Bruker, 2021 ▸), *SAINT* (Bruker, 2021 ▸), *SHELXT2014* (Sheldrick, 2015*a* ▸), *SHELXS2014* (Sheldrick, 2015*a* ▸), *SHELXL2018* (Sheldrick, 2015*b* ▸), and *OLEX2* (Dolomanov *et al.*, 2009 ▸).

## supplementary crystallographic information

### 4-Phenoxy-1H-indole (1) Crystal data

**Table:**

C_15_H_13_NO	*D*_x_ = 1.242 Mg m^−^^3^
*M**_r_* = 223.26	Mo *K*α radiation, λ = 0.71073 Å
Orthorhombic, *P**b**c**a*	Cell parameters from 9827 reflections
*a* = 9.8201 (9) Å	θ = 2.6–26.3°
*b* = 9.0067 (7) Å	µ = 0.08 mm^−^^1^
*c* = 26.995 (2) Å	*T* = 300 K
*V* = 2387.6 (4) Å^3^	Block, colourless
*Z* = 8	0.40 × 0.22 × 0.12 mm
*F*(000) = 944	

### 4-Phenoxy-1H-indole (1) Data collection

**Table:**

Bruker D8 Venture CMOS diffractometer	2098 reflections with *I* > 2σ(*I*)
φ and ω scans	*R*_int_ = 0.039
Absorption correction: multi-scan (SADABS; Bruker, 2021)	θ_max_ = 26.4°, θ_min_ = 3.0°
*T*_min_ = 0.697, *T*_max_ = 0.745	*h* = −12→12
56335 measured reflections	*k* = −11→10
2451 independent reflections	*l* = −33→33

### 4-Phenoxy-1H-indole (1) Refinement

**Table:**

Refinement on *F*^2^	Hydrogen site location: mixed
Least-squares matrix: full	H atoms treated by a mixture of independent and constrained refinement
*R*[*F*^2^ > 2σ(*F*^2^)] = 0.045	*w* = 1/[σ^2^(*F*_o_^2^) + (0.0359*P*)^2^ + 0.787*P*] where *P* = (*F*_o_^2^ + 2*F*_c_^2^)/3
*wR*(*F*^2^) = 0.110	(Δ/σ)_max_ < 0.001
*S* = 1.11	Δρ_max_ = 0.16 e Å^−^^3^
2451 reflections	Δρ_min_ = −0.12 e Å^−^^3^
159 parameters	Extinction correction: SHELXL-2018/3 (Sheldrick 2015b), Fc^*^=kFc[1+0.001xFc^2^λ^3^/sin(2θ)]^-1/4^
0 restraints	Extinction coefficient: 0.0078 (11)

### 4-Phenoxy-1H-indole (1) Special details

**Table:**

Geometry. All esds (except the esd in the dihedral angle between two l.s. planes) are estimated using the full covariance matrix. The cell esds are taken into account individually in the estimation of esds in distances, angles and torsion angles; correlations between esds in cell parameters are only used when they are defined by crystal symmetry. An approximate (isotropic) treatment of cell esds is used for estimating esds involving l.s. planes.

### 4-Phenoxy-1H-indole (1) Fractional atomic coordinates and isotropic or equivalent isotropic displacement parameters (Å^2^)

**Table:**

	*x*	*y*	*z*	*U*_iso_*/*U*_eq_	
O1	0.45181 (11)	0.35485 (11)	0.37604 (4)	0.0502 (3)	
N1	0.64192 (16)	−0.06802 (16)	0.31310 (6)	0.0608 (4)	
H1	0.659 (2)	−0.148 (2)	0.2961 (8)	0.081 (6)*	
C1	0.71754 (19)	−0.01437 (19)	0.35146 (7)	0.0602 (5)	
H1A	0.796959	−0.057322	0.363578	0.072*	
C2	0.53303 (16)	0.02425 (16)	0.30472 (6)	0.0494 (4)	
C3	0.43121 (19)	0.01880 (19)	0.26876 (6)	0.0599 (5)	
H3	0.428870	−0.056114	0.245062	0.072*	
C4	0.33512 (18)	0.1283 (2)	0.26987 (6)	0.0600 (5)	
H4	0.266037	0.127455	0.246312	0.072*	
C5	0.33709 (16)	0.24214 (18)	0.30539 (6)	0.0511 (4)	
H5	0.269033	0.313937	0.305279	0.061*	
C6	0.43895 (15)	0.24842 (15)	0.34041 (5)	0.0430 (3)	
C7	0.54041 (15)	0.13840 (15)	0.34020 (5)	0.0428 (3)	
C8	0.65916 (17)	0.11138 (17)	0.36927 (6)	0.0506 (4)	
H8	0.690629	0.169017	0.395459	0.061*	
C9	0.34925 (19)	0.4685 (2)	0.37747 (7)	0.0668 (5)	
H9A	0.260551	0.424643	0.383434	0.080*	
H9B	0.346282	0.520817	0.346073	0.080*	
C10	0.38433 (16)	0.57386 (17)	0.41830 (6)	0.0523 (4)	
C11	0.3366 (2)	0.5527 (2)	0.46590 (7)	0.0689 (5)	
H11	0.278860	0.473301	0.472565	0.083*	
C12	0.3732 (2)	0.6474 (3)	0.50358 (8)	0.0809 (6)	
H12	0.340519	0.631075	0.535449	0.097*	
C13	0.4566 (2)	0.7645 (2)	0.49464 (8)	0.0759 (6)	
H13	0.481679	0.827876	0.520269	0.091*	
C14	0.5032 (2)	0.7883 (2)	0.44799 (9)	0.0790 (6)	
H14	0.559340	0.869157	0.441610	0.095*	
C15	0.4679 (2)	0.6935 (2)	0.40992 (7)	0.0669 (5)	
H15	0.501146	0.710903	0.378193	0.080*	

### 4-Phenoxy-1H-indole (1) Atomic displacement parameters (Å^2^)

**Table:**

	*U*^11^	*U*^22^	*U*^33^	*U*^12^	*U*^13^	*U*^23^
O1	0.0511 (6)	0.0444 (6)	0.0550 (6)	0.0090 (5)	−0.0092 (5)	−0.0083 (5)
N1	0.0713 (10)	0.0414 (7)	0.0696 (9)	0.0005 (7)	0.0253 (8)	−0.0041 (7)
C1	0.0624 (11)	0.0513 (9)	0.0669 (10)	0.0107 (8)	0.0129 (9)	0.0111 (8)
C2	0.0548 (9)	0.0420 (8)	0.0514 (8)	−0.0100 (7)	0.0189 (7)	0.0003 (7)
C3	0.0697 (11)	0.0574 (10)	0.0526 (9)	−0.0223 (9)	0.0118 (8)	−0.0129 (8)
C4	0.0618 (10)	0.0669 (11)	0.0513 (9)	−0.0211 (9)	−0.0041 (8)	−0.0027 (8)
C5	0.0496 (9)	0.0509 (9)	0.0528 (9)	−0.0062 (7)	−0.0035 (7)	0.0030 (7)
C6	0.0475 (8)	0.0387 (7)	0.0429 (7)	−0.0056 (6)	0.0029 (6)	0.0016 (6)
C7	0.0478 (8)	0.0381 (7)	0.0426 (7)	−0.0050 (6)	0.0080 (6)	0.0037 (6)
C8	0.0556 (9)	0.0444 (8)	0.0519 (9)	0.0039 (7)	0.0035 (7)	0.0052 (7)
C9	0.0596 (11)	0.0601 (10)	0.0808 (12)	0.0192 (9)	−0.0176 (9)	−0.0165 (9)
C10	0.0465 (8)	0.0466 (8)	0.0636 (10)	0.0119 (7)	−0.0016 (7)	−0.0060 (7)
C11	0.0643 (11)	0.0618 (11)	0.0806 (12)	−0.0071 (9)	0.0135 (10)	−0.0012 (10)
C12	0.0892 (15)	0.0908 (15)	0.0627 (11)	0.0035 (13)	0.0165 (11)	−0.0105 (11)
C13	0.0741 (13)	0.0727 (13)	0.0809 (13)	0.0049 (11)	−0.0008 (11)	−0.0264 (11)
C14	0.0742 (13)	0.0569 (11)	0.1059 (16)	−0.0140 (10)	0.0105 (12)	−0.0099 (11)
C15	0.0702 (12)	0.0633 (11)	0.0673 (11)	−0.0006 (9)	0.0149 (9)	0.0001 (9)

### 4-Phenoxy-1H-indole (1) Geometric parameters (Å, º)

**Table:**

O1—C6	1.3636 (17)	C7—C8	1.426 (2)
O1—C9	1.4368 (19)	C8—H8	0.9300
N1—H1	0.87 (2)	C9—H9A	0.9700
N1—C1	1.363 (2)	C9—H9B	0.9700
N1—C2	1.373 (2)	C9—C10	1.494 (2)
C1—H1A	0.9300	C10—C11	1.381 (2)
C1—C8	1.357 (2)	C10—C15	1.373 (2)
C2—C3	1.394 (2)	C11—H11	0.9300
C2—C7	1.407 (2)	C11—C12	1.375 (3)
C3—H3	0.9300	C12—H12	0.9300
C3—C4	1.365 (3)	C12—C13	1.357 (3)
C4—H4	0.9300	C13—H13	0.9300
C4—C5	1.404 (2)	C13—C14	1.357 (3)
C5—H5	0.9300	C14—H14	0.9300
C5—C6	1.377 (2)	C14—C15	1.380 (3)
C6—C7	1.405 (2)	C15—H15	0.9300
			
C6—O1—C9	117.07 (12)	C1—C8—H8	126.5
C1—N1—H1	125.9 (14)	C7—C8—H8	126.5
C1—N1—C2	109.56 (14)	O1—C9—H9A	110.1
C2—N1—H1	124.5 (14)	O1—C9—H9B	110.1
N1—C1—H1A	125.2	O1—C9—C10	108.10 (13)
C8—C1—N1	109.56 (16)	H9A—C9—H9B	108.4
C8—C1—H1A	125.2	C10—C9—H9A	110.1
N1—C2—C3	130.67 (15)	C10—C9—H9B	110.1
N1—C2—C7	106.86 (14)	C11—C10—C9	121.35 (17)
C3—C2—C7	122.45 (15)	C15—C10—C9	120.96 (16)
C2—C3—H3	121.5	C15—C10—C11	117.68 (16)
C4—C3—C2	117.05 (15)	C10—C11—H11	119.5
C4—C3—H3	121.5	C12—C11—C10	120.90 (18)
C3—C4—H4	118.9	C12—C11—H11	119.5
C3—C4—C5	122.22 (16)	C11—C12—H12	119.7
C5—C4—H4	118.9	C13—C12—C11	120.56 (19)
C4—C5—H5	119.7	C13—C12—H12	119.7
C6—C5—C4	120.58 (16)	C12—C13—H13	120.3
C6—C5—H5	119.7	C14—C13—C12	119.40 (19)
O1—C6—C5	125.45 (14)	C14—C13—H13	120.3
O1—C6—C7	115.65 (13)	C13—C14—H14	119.7
C5—C6—C7	118.89 (14)	C13—C14—C15	120.61 (19)
C2—C7—C8	106.97 (14)	C15—C14—H14	119.7
C6—C7—C2	118.78 (14)	C10—C15—C14	120.84 (17)
C6—C7—C8	134.25 (14)	C10—C15—H15	119.6
C1—C8—C7	107.03 (15)	C14—C15—H15	119.6
			
O1—C6—C7—C2	179.47 (12)	C4—C5—C6—C7	−0.4 (2)
O1—C6—C7—C8	−0.8 (2)	C5—C6—C7—C2	−1.1 (2)
O1—C9—C10—C11	−90.2 (2)	C5—C6—C7—C8	178.69 (15)
O1—C9—C10—C15	88.3 (2)	C6—O1—C9—C10	−179.00 (13)
N1—C1—C8—C7	−0.28 (18)	C6—C7—C8—C1	179.77 (16)
N1—C2—C3—C4	−179.92 (15)	C7—C2—C3—C4	−1.5 (2)
N1—C2—C7—C6	−179.18 (12)	C9—O1—C6—C5	0.9 (2)
N1—C2—C7—C8	1.00 (15)	C9—O1—C6—C7	−179.63 (14)
C1—N1—C2—C3	177.39 (15)	C9—C10—C11—C12	177.77 (18)
C1—N1—C2—C7	−1.20 (17)	C9—C10—C15—C14	−178.20 (17)
C2—N1—C1—C8	0.94 (18)	C10—C11—C12—C13	0.4 (3)
C2—C3—C4—C5	0.0 (2)	C11—C10—C15—C14	0.4 (3)
C2—C7—C8—C1	−0.45 (16)	C11—C12—C13—C14	0.5 (3)
C3—C2—C7—C6	2.1 (2)	C12—C13—C14—C15	−0.9 (3)
C3—C2—C7—C8	−177.73 (14)	C13—C14—C15—C10	0.5 (3)
C3—C4—C5—C6	1.0 (2)	C15—C10—C11—C12	−0.8 (3)
C4—C5—C6—O1	179.00 (14)		

### 4-Phenoxy-1H-indole (1) Hydrogen-bond geometry (Å, º)

**Table:**

*D*—H···*A*	*D*—H	H···*A*	*D*···*A*	*D*—H···*A*
C1—H1*A*···O1^i^	0.93	2.61	3.517 (2)	164

Symmetry code: (i) −*x*+3/2, *y*−1/2, *z*.

### 2-[4-(Benzyloxy)-1H-indol-3-yl]-2-oxo-N-(propan-2-yl)acetamide (2) Crystal data

**Table:**

C_20_H_20_N_2_O_3_	*D*_x_ = 1.239 Mg m^−^^3^
*M**_r_* = 336.38	Mo *K*α radiation, λ = 0.71073 Å
Orthorhombic, *P**n**a*2_1_	Cell parameters from 9851 reflections
*a* = 16.4230 (13) Å	θ = 2.6–23.9°
*b* = 6.5609 (4) Å	µ = 0.08 mm^−^^1^
*c* = 33.469 (2) Å	*T* = 300 K
*V* = 3606.2 (4) Å^3^	Block, brown
*Z* = 8	0.34 × 0.30 × 0.08 mm
*F*(000) = 1424	

### 2-[4-(Benzyloxy)-1H-indol-3-yl]-2-oxo-N-(propan-2-yl)acetamide (2) Data collection

**Table:**

Bruker D8 Venture CMOS diffractometer	5295 reflections with *I* > 2σ(*I*)
φ and ω scans	*R*_int_ = 0.037
Absorption correction: multi-scan (SADABS; Bruker, 2021)	θ_max_ = 25.4°, θ_min_ = 2.6°
*T*_min_ = 0.694, *T*_max_ = 0.745	*h* = −19→19
48212 measured reflections	*k* = −7→7
6584 independent reflections	*l* = −40→40

### 2-[4-(Benzyloxy)-1H-indol-3-yl]-2-oxo-N-(propan-2-yl)acetamide (2) Refinement

**Table:**

Refinement on *F*^2^	Hydrogen site location: mixed
Least-squares matrix: full	H atoms treated by a mixture of independent and constrained refinement
*R*[*F*^2^ > 2σ(*F*^2^)] = 0.046	*w* = 1/[σ^2^(*F*_o_^2^) + (0.0416*P*)^2^ + 1.3827*P*] where *P* = (*F*_o_^2^ + 2*F*_c_^2^)/3
*wR*(*F*^2^) = 0.117	(Δ/σ)_max_ < 0.001
*S* = 1.04	Δρ_max_ = 0.17 e Å^−^^3^
6584 reflections	Δρ_min_ = −0.15 e Å^−^^3^
468 parameters	Absolute structure: Refined as an inversion twin
4 restraints	Absolute structure parameter: 0.1 (18)

### 2-[4-(Benzyloxy)-1H-indol-3-yl]-2-oxo-N-(propan-2-yl)acetamide (2) Special details

**Table:**

Geometry. All esds (except the esd in the dihedral angle between two l.s. planes) are estimated using the full covariance matrix. The cell esds are taken into account individually in the estimation of esds in distances, angles and torsion angles; correlations between esds in cell parameters are only used when they are defined by crystal symmetry. An approximate (isotropic) treatment of cell esds is used for estimating esds involving l.s. planes.
Refinement. Refined as a 2-component inversion twin.

### 2-[4-(Benzyloxy)-1H-indol-3-yl]-2-oxo-N-(propan-2-yl)acetamide (2) Fractional atomic coordinates and isotropic or equivalent isotropic displacement parameters (Å^2^)

**Table:**

	*x*	*y*	*z*	*U*_iso_*/*U*_eq_	
O1	0.5240 (2)	0.4291 (5)	0.63818 (8)	0.0793 (10)	
O2	0.4162 (2)	0.2561 (6)	0.55613 (9)	0.0768 (10)	
O3	0.58588 (18)	0.8372 (5)	0.63145 (7)	0.0597 (8)	
N1	0.6357 (2)	0.4857 (7)	0.51462 (11)	0.0679 (11)	
H1	0.654322	0.447962	0.491765	0.081*	
N2	0.4109 (2)	0.1572 (6)	0.62014 (11)	0.0657 (10)	
C1	0.5799 (3)	0.3846 (8)	0.53570 (13)	0.0636 (11)	
H1A	0.554698	0.264721	0.527535	0.076*	
C2	0.6597 (3)	0.6615 (8)	0.53495 (12)	0.0589 (11)	
C3	0.7126 (3)	0.8123 (10)	0.52259 (15)	0.0748 (15)	
H3	0.739396	0.805187	0.498151	0.090*	
C4	0.7236 (3)	0.9720 (9)	0.54802 (14)	0.0741 (14)	
H4	0.758745	1.076869	0.540825	0.089*	
C5	0.6830 (3)	0.9818 (8)	0.58479 (12)	0.0612 (11)	
H5	0.692192	1.092406	0.601560	0.073*	
C6	0.6296 (2)	0.8310 (6)	0.59669 (11)	0.0492 (10)	
C7	0.6170 (2)	0.6621 (6)	0.57142 (11)	0.0487 (9)	
C8	0.5648 (3)	0.4837 (6)	0.57149 (11)	0.0502 (9)	
C9	0.5135 (3)	0.4002 (6)	0.60271 (11)	0.0523 (10)	
C10	0.4423 (3)	0.2625 (6)	0.59046 (12)	0.0538 (10)	
C11	0.3443 (3)	0.0112 (7)	0.61586 (14)	0.0683 (12)	
H11	0.310596	0.052903	0.593100	0.082*	
C12	0.2927 (4)	0.0139 (12)	0.6525 (2)	0.120 (3)	
H12A	0.246303	−0.072908	0.648646	0.180*	
H12B	0.274682	0.150566	0.657642	0.180*	
H12C	0.323935	−0.034373	0.674832	0.180*	
C13	0.3778 (4)	−0.1934 (10)	0.6075 (3)	0.137 (3)	
H13A	0.333875	−0.287603	0.603218	0.205*	
H13B	0.409957	−0.237864	0.629772	0.205*	
H13C	0.411226	−0.187746	0.583979	0.205*	
C14	0.6120 (3)	0.9724 (7)	0.66161 (12)	0.0606 (11)	
H14A	0.611897	1.110978	0.651536	0.073*	
H14B	0.667060	0.938485	0.669739	0.073*	
C15	0.5556 (3)	0.9555 (7)	0.69666 (12)	0.0569 (10)	
C16	0.5345 (3)	1.1282 (8)	0.71718 (14)	0.0739 (13)	
H16	0.554437	1.254255	0.709074	0.089*	
C17	0.4829 (4)	1.1146 (11)	0.75046 (16)	0.0929 (19)	
H17	0.468398	1.231677	0.764431	0.112*	
C18	0.4540 (4)	0.9305 (12)	0.76219 (16)	0.0919 (18)	
H18	0.420685	0.921799	0.784579	0.110*	
C19	0.4728 (4)	0.7627 (11)	0.74217 (16)	0.0945 (19)	
H19	0.452191	0.637347	0.750308	0.113*	
C20	0.5232 (4)	0.7744 (9)	0.70914 (17)	0.0851 (16)	
H20	0.535284	0.656109	0.695042	0.102*	
O4	0.2897 (2)	0.4332 (5)	0.36366 (8)	0.0784 (10)	
O5	0.1974 (2)	0.2158 (6)	0.44698 (10)	0.0901 (11)	
O6	0.35700 (18)	0.8328 (5)	0.36871 (8)	0.0625 (8)	
N3	0.4045 (3)	0.4959 (7)	0.48712 (12)	0.0727 (12)	
N4	0.1780 (3)	0.1631 (6)	0.38136 (12)	0.0727 (11)	
C21	0.3495 (3)	0.3903 (8)	0.46630 (13)	0.0644 (12)	
H21	0.324853	0.270395	0.474817	0.077*	
C22	0.4279 (3)	0.6671 (8)	0.46657 (13)	0.0617 (12)	
C23	0.4817 (3)	0.8206 (10)	0.47795 (14)	0.0741 (14)	
H23	0.507633	0.817635	0.502655	0.089*	
C24	0.4950 (3)	0.9755 (9)	0.45151 (15)	0.0779 (14)	
H24	0.531739	1.078188	0.458057	0.093*	
C25	0.4543 (3)	0.9828 (8)	0.41458 (13)	0.0658 (12)	
H25	0.464119	1.090643	0.397189	0.079*	
C26	0.4000 (3)	0.8317 (7)	0.40384 (12)	0.0537 (10)	
C27	0.3857 (2)	0.6662 (7)	0.42979 (11)	0.0521 (10)	
C28	0.3347 (3)	0.4858 (7)	0.43015 (11)	0.0557 (11)	
C29	0.2838 (3)	0.3987 (7)	0.39941 (12)	0.0602 (11)	
C30	0.2157 (3)	0.2463 (7)	0.41220 (13)	0.0630 (12)	
C31	0.1127 (3)	0.0105 (8)	0.38476 (16)	0.0762 (14)	
H31	0.079731	0.044781	0.408215	0.091*	
C32	0.1477 (4)	−0.1950 (10)	0.3911 (3)	0.132 (3)	
H32A	0.105164	−0.288177	0.398326	0.198*	
H32B	0.187428	−0.189390	0.412091	0.198*	
H32C	0.173287	−0.240602	0.366869	0.198*	
C33	0.0584 (4)	0.0240 (12)	0.3481 (2)	0.118 (2)	
H33A	0.014535	−0.071859	0.350550	0.176*	
H33B	0.089728	−0.007008	0.324646	0.176*	
H33C	0.036669	0.159366	0.345916	0.176*	
C34	0.3794 (3)	0.9784 (7)	0.33933 (13)	0.0627 (11)	
H34A	0.435266	0.955549	0.331010	0.075*	
H34B	0.375553	1.114783	0.350360	0.075*	
C35	0.3235 (3)	0.9582 (7)	0.30415 (12)	0.0569 (10)	
C36	0.2928 (4)	0.7746 (8)	0.29169 (16)	0.0822 (16)	
H36	0.305948	0.656060	0.305515	0.099*	
C37	0.2430 (4)	0.7651 (11)	0.25894 (19)	0.102 (2)	
H37	0.223112	0.639073	0.250766	0.122*	
C38	0.2219 (4)	0.9337 (13)	0.23815 (16)	0.0936 (19)	
H38	0.188330	0.924009	0.215841	0.112*	
C39	0.2504 (4)	1.1171 (10)	0.25040 (16)	0.0881 (17)	
H39	0.235846	1.234867	0.236685	0.106*	
C40	0.3017 (3)	1.1296 (8)	0.28350 (14)	0.0709 (13)	
H40	0.321249	1.255985	0.291639	0.085*	
H2	0.433 (2)	0.187 (7)	0.6439 (7)	0.057 (12)*	
H4A	0.199 (3)	0.184 (8)	0.3569 (8)	0.091 (17)*	
H3A	0.420 (3)	0.448 (7)	0.5101 (8)	0.086 (16)*	

### 2-[4-(Benzyloxy)-1H-indol-3-yl]-2-oxo-N-(propan-2-yl)acetamide (2) Atomic displacement parameters (Å^2^)

**Table:**

	*U*^11^	*U*^22^	*U*^33^	*U*^12^	*U*^13^	*U*^23^
O1	0.131 (3)	0.071 (2)	0.0366 (16)	−0.029 (2)	−0.0051 (17)	0.0044 (14)
O2	0.091 (2)	0.097 (2)	0.0430 (17)	−0.019 (2)	−0.0148 (16)	0.0155 (16)
O3	0.0653 (17)	0.0731 (19)	0.0406 (15)	−0.0187 (15)	0.0093 (13)	−0.0113 (14)
N1	0.065 (2)	0.094 (3)	0.045 (2)	0.013 (2)	0.0100 (18)	−0.014 (2)
N2	0.082 (3)	0.070 (2)	0.046 (2)	−0.016 (2)	−0.0082 (19)	0.0055 (18)
C1	0.067 (3)	0.072 (3)	0.051 (2)	0.011 (2)	0.000 (2)	−0.010 (2)
C2	0.049 (2)	0.084 (3)	0.043 (2)	0.013 (2)	0.0008 (19)	−0.002 (2)
C3	0.056 (3)	0.115 (4)	0.054 (3)	−0.001 (3)	0.017 (2)	0.001 (3)
C4	0.059 (3)	0.101 (4)	0.063 (3)	−0.012 (3)	0.015 (2)	0.008 (3)
C5	0.058 (3)	0.071 (3)	0.055 (3)	−0.004 (2)	0.009 (2)	0.002 (2)
C6	0.044 (2)	0.064 (3)	0.040 (2)	0.0053 (19)	0.0027 (17)	0.0007 (19)
C7	0.045 (2)	0.066 (3)	0.035 (2)	0.0082 (19)	−0.0007 (16)	0.0049 (18)
C8	0.057 (2)	0.059 (2)	0.0351 (18)	0.010 (2)	−0.0030 (17)	−0.0009 (18)
C9	0.078 (3)	0.044 (2)	0.035 (2)	0.002 (2)	−0.0022 (19)	0.0018 (17)
C10	0.069 (3)	0.050 (2)	0.043 (2)	0.006 (2)	−0.0006 (19)	0.0052 (17)
C11	0.071 (3)	0.076 (3)	0.058 (3)	−0.013 (2)	−0.005 (2)	0.006 (2)
C12	0.119 (5)	0.144 (6)	0.096 (5)	−0.051 (5)	0.019 (4)	−0.003 (4)
C13	0.097 (5)	0.082 (4)	0.232 (10)	−0.016 (4)	−0.025 (6)	−0.044 (5)
C14	0.060 (3)	0.069 (3)	0.052 (3)	−0.004 (2)	0.004 (2)	−0.009 (2)
C15	0.056 (2)	0.070 (3)	0.045 (2)	0.000 (2)	−0.0023 (19)	−0.006 (2)
C16	0.099 (4)	0.074 (3)	0.049 (3)	0.012 (3)	−0.003 (2)	−0.001 (2)
C17	0.116 (5)	0.109 (5)	0.053 (3)	0.048 (4)	0.005 (3)	−0.015 (3)
C18	0.083 (4)	0.137 (6)	0.056 (3)	0.000 (4)	0.013 (3)	0.006 (4)
C19	0.125 (5)	0.104 (5)	0.054 (3)	−0.039 (4)	0.016 (3)	−0.003 (3)
C20	0.106 (4)	0.082 (4)	0.068 (3)	−0.022 (3)	0.014 (3)	−0.017 (3)
O4	0.129 (3)	0.0681 (19)	0.0376 (16)	−0.025 (2)	0.0117 (17)	0.0062 (15)
O5	0.102 (3)	0.117 (3)	0.051 (2)	−0.014 (2)	0.0224 (18)	0.0188 (19)
O6	0.0669 (19)	0.0725 (19)	0.0483 (17)	−0.0109 (15)	−0.0070 (13)	0.0182 (14)
N3	0.070 (2)	0.102 (3)	0.046 (2)	0.028 (2)	0.005 (2)	0.016 (2)
N4	0.093 (3)	0.073 (3)	0.051 (2)	−0.010 (2)	0.015 (2)	0.009 (2)
C21	0.068 (3)	0.078 (3)	0.048 (2)	0.019 (2)	0.012 (2)	0.014 (2)
C22	0.058 (3)	0.085 (3)	0.042 (2)	0.023 (2)	0.008 (2)	0.007 (2)
C23	0.069 (3)	0.109 (4)	0.045 (2)	0.011 (3)	−0.009 (2)	−0.009 (3)
C24	0.078 (3)	0.093 (4)	0.064 (3)	0.002 (3)	−0.010 (3)	−0.014 (3)
C25	0.068 (3)	0.072 (3)	0.058 (3)	0.001 (2)	0.000 (2)	−0.004 (2)
C26	0.055 (2)	0.063 (3)	0.043 (2)	0.009 (2)	0.0028 (18)	−0.0016 (19)
C27	0.052 (2)	0.066 (3)	0.039 (2)	0.017 (2)	0.0084 (17)	0.0039 (19)
C28	0.061 (3)	0.066 (3)	0.040 (2)	0.017 (2)	0.0141 (19)	0.0084 (19)
C29	0.081 (3)	0.055 (2)	0.044 (2)	0.013 (2)	0.017 (2)	0.007 (2)
C30	0.082 (3)	0.066 (3)	0.041 (2)	0.012 (3)	0.013 (2)	0.012 (2)
C31	0.073 (3)	0.084 (3)	0.071 (3)	−0.009 (3)	0.018 (3)	0.013 (3)
C32	0.101 (5)	0.079 (4)	0.215 (9)	−0.015 (4)	0.027 (5)	0.038 (5)
C33	0.115 (5)	0.131 (6)	0.107 (5)	−0.024 (5)	−0.019 (4)	0.013 (4)
C34	0.071 (3)	0.064 (3)	0.053 (3)	−0.005 (2)	0.003 (2)	0.012 (2)
C35	0.060 (3)	0.067 (3)	0.043 (2)	−0.003 (2)	0.0055 (19)	0.012 (2)
C36	0.113 (4)	0.076 (3)	0.058 (3)	−0.023 (3)	−0.012 (3)	0.016 (3)
C37	0.115 (5)	0.110 (5)	0.080 (4)	−0.043 (4)	−0.022 (4)	0.006 (3)
C38	0.085 (4)	0.142 (6)	0.054 (3)	0.006 (4)	−0.007 (3)	0.004 (4)
C39	0.112 (4)	0.095 (4)	0.058 (3)	0.037 (4)	−0.006 (3)	0.012 (3)
C40	0.094 (4)	0.065 (3)	0.054 (3)	0.014 (3)	0.002 (2)	0.004 (2)

### 2-[4-(Benzyloxy)-1H-indol-3-yl]-2-oxo-N-(propan-2-yl)acetamide (2) Geometric parameters (Å, º)

**Table:**

O1—C9	1.215 (4)	O4—C29	1.222 (5)
O2—C10	1.227 (5)	O5—C30	1.219 (5)
O3—C6	1.368 (4)	O6—C26	1.372 (5)
O3—C14	1.411 (5)	O6—C34	1.420 (5)
N1—H1	0.8600	N3—C21	1.334 (6)
N1—C1	1.333 (6)	N3—C22	1.372 (7)
N1—C2	1.396 (6)	N3—H3A	0.871 (15)
N2—C10	1.315 (5)	N4—C30	1.322 (6)
N2—C11	1.461 (6)	N4—C31	1.471 (6)
N2—H2	0.894 (14)	N4—H4A	0.897 (15)
C1—H1A	0.9300	C21—H21	0.9300
C1—C8	1.385 (6)	C21—C28	1.384 (6)
C2—C3	1.381 (7)	C22—C23	1.394 (8)
C2—C7	1.408 (5)	C22—C27	1.412 (6)
C3—H3	0.9300	C23—H23	0.9300
C3—C4	1.362 (8)	C23—C24	1.365 (8)
C4—H4	0.9300	C24—H24	0.9300
C4—C5	1.401 (6)	C24—C25	1.406 (6)
C5—H5	0.9300	C25—H25	0.9300
C5—C6	1.380 (6)	C25—C26	1.381 (7)
C6—C7	1.410 (6)	C26—C27	1.410 (6)
C7—C8	1.451 (6)	C27—C28	1.450 (6)
C8—C9	1.450 (6)	C28—C29	1.444 (6)
C9—C10	1.533 (6)	C29—C30	1.560 (6)
C11—H11	0.9800	C31—H31	0.9800
C11—C12	1.491 (8)	C31—C32	1.481 (8)
C11—C13	1.478 (8)	C31—C33	1.520 (8)
C12—H12A	0.9600	C32—H32A	0.9600
C12—H12B	0.9600	C32—H32B	0.9600
C12—H12C	0.9600	C32—H32C	0.9600
C13—H13A	0.9600	C33—H33A	0.9600
C13—H13B	0.9600	C33—H33B	0.9600
C13—H13C	0.9600	C33—H33C	0.9600
C14—H14A	0.9700	C34—H34A	0.9700
C14—H14B	0.9700	C34—H34B	0.9700
C14—C15	1.499 (6)	C34—C35	1.499 (6)
C15—C16	1.369 (7)	C35—C36	1.371 (7)
C15—C20	1.367 (7)	C35—C40	1.368 (6)
C16—H16	0.9300	C36—H36	0.9300
C16—C17	1.403 (8)	C36—C37	1.369 (8)
C17—H17	0.9300	C37—H37	0.9300
C17—C18	1.356 (9)	C37—C38	1.352 (9)
C18—H18	0.9300	C38—H38	0.9300
C18—C19	1.325 (9)	C38—C39	1.355 (9)
C19—H19	0.9300	C39—H39	0.9300
C19—C20	1.382 (8)	C39—C40	1.393 (7)
C20—H20	0.9300	C40—H40	0.9300
			
C6—O3—C14	117.9 (3)	C26—O6—C34	117.6 (3)
C1—N1—H1	124.9	C21—N3—C22	110.6 (4)
C1—N1—C2	110.3 (4)	C21—N3—H3A	119 (3)
C2—N1—H1	124.9	C22—N3—H3A	131 (3)
C10—N2—C11	124.3 (4)	C30—N4—C31	124.2 (4)
C10—N2—H2	113 (3)	C30—N4—H4A	118 (4)
C11—N2—H2	122 (3)	C31—N4—H4A	117 (4)
N1—C1—H1A	124.8	N3—C21—H21	125.0
N1—C1—C8	110.3 (4)	N3—C21—C28	109.9 (5)
C8—C1—H1A	124.8	C28—C21—H21	125.0
N1—C2—C7	106.6 (4)	N3—C22—C23	129.2 (5)
C3—C2—N1	128.6 (4)	N3—C22—C27	107.2 (4)
C3—C2—C7	124.8 (5)	C23—C22—C27	123.5 (4)
C2—C3—H3	121.7	C22—C23—H23	121.2
C4—C3—C2	116.6 (4)	C24—C23—C22	117.5 (4)
C4—C3—H3	121.7	C24—C23—H23	121.2
C3—C4—H4	119.3	C23—C24—H24	119.4
C3—C4—C5	121.4 (5)	C23—C24—C25	121.3 (5)
C5—C4—H4	119.3	C25—C24—H24	119.4
C4—C5—H5	119.2	C24—C25—H25	119.6
C6—C5—C4	121.5 (4)	C26—C25—C24	120.8 (5)
C6—C5—H5	119.2	C26—C25—H25	119.6
O3—C6—C5	123.9 (4)	O6—C26—C25	123.5 (4)
O3—C6—C7	117.1 (4)	O6—C26—C27	116.5 (4)
C5—C6—C7	118.9 (4)	C25—C26—C27	120.0 (4)
C2—C7—C6	116.7 (4)	C22—C27—C28	106.2 (4)
C2—C7—C8	107.0 (4)	C26—C27—C22	116.9 (4)
C6—C7—C8	136.1 (3)	C26—C27—C28	136.9 (4)
C1—C8—C7	105.8 (4)	C21—C28—C27	106.0 (4)
C1—C8—C9	123.4 (4)	C21—C28—C29	123.1 (4)
C9—C8—C7	130.5 (3)	C29—C28—C27	130.7 (4)
O1—C9—C8	124.3 (4)	O4—C29—C28	125.4 (4)
O1—C9—C10	117.5 (4)	O4—C29—C30	116.4 (4)
C8—C9—C10	118.2 (3)	C28—C29—C30	118.3 (3)
O2—C10—N2	123.5 (4)	O5—C30—N4	124.2 (5)
O2—C10—C9	122.5 (4)	O5—C30—C29	123.0 (4)
N2—C10—C9	114.0 (4)	N4—C30—C29	112.7 (4)
N2—C11—H11	108.4	N4—C31—H31	108.0
N2—C11—C12	109.7 (4)	N4—C31—C32	110.3 (5)
N2—C11—C13	109.6 (4)	N4—C31—C33	109.0 (4)
C12—C11—H11	108.4	C32—C31—H31	108.0
C13—C11—H11	108.4	C32—C31—C33	113.3 (6)
C13—C11—C12	112.2 (6)	C33—C31—H31	108.0
C11—C12—H12A	109.5	C31—C32—H32A	109.5
C11—C12—H12B	109.5	C31—C32—H32B	109.5
C11—C12—H12C	109.5	C31—C32—H32C	109.5
H12A—C12—H12B	109.5	H32A—C32—H32B	109.5
H12A—C12—H12C	109.5	H32A—C32—H32C	109.5
H12B—C12—H12C	109.5	H32B—C32—H32C	109.5
C11—C13—H13A	109.5	C31—C33—H33A	109.5
C11—C13—H13B	109.5	C31—C33—H33B	109.5
C11—C13—H13C	109.5	C31—C33—H33C	109.5
H13A—C13—H13B	109.5	H33A—C33—H33B	109.5
H13A—C13—H13C	109.5	H33A—C33—H33C	109.5
H13B—C13—H13C	109.5	H33B—C33—H33C	109.5
O3—C14—H14A	109.9	O6—C34—H34A	109.9
O3—C14—H14B	109.9	O6—C34—H34B	109.9
O3—C14—C15	109.0 (3)	O6—C34—C35	109.0 (3)
H14A—C14—H14B	108.3	H34A—C34—H34B	108.3
C15—C14—H14A	109.9	C35—C34—H34A	109.9
C15—C14—H14B	109.9	C35—C34—H34B	109.9
C16—C15—C14	119.2 (4)	C36—C35—C34	122.8 (4)
C20—C15—C14	122.9 (4)	C40—C35—C34	119.0 (4)
C20—C15—C16	117.9 (4)	C40—C35—C36	118.2 (4)
C15—C16—H16	120.1	C35—C36—H36	119.9
C15—C16—C17	119.9 (5)	C37—C36—C35	120.2 (5)
C17—C16—H16	120.1	C37—C36—H36	119.9
C16—C17—H17	120.1	C36—C37—H37	119.1
C18—C17—C16	119.8 (5)	C38—C37—C36	121.9 (6)
C18—C17—H17	120.1	C38—C37—H37	119.1
C17—C18—H18	119.6	C37—C38—H38	120.6
C19—C18—C17	120.8 (5)	C37—C38—C39	118.8 (5)
C19—C18—H18	119.6	C39—C38—H38	120.6
C18—C19—H19	120.1	C38—C39—H39	119.9
C18—C19—C20	119.8 (6)	C38—C39—C40	120.1 (5)
C20—C19—H19	120.1	C40—C39—H39	119.9
C15—C20—C19	121.7 (5)	C35—C40—C39	120.8 (5)
C15—C20—H20	119.1	C35—C40—H40	119.6
C19—C20—H20	119.1	C39—C40—H40	119.6
			
O1—C9—C10—O2	164.9 (5)	O4—C29—C30—O5	−171.7 (5)
O1—C9—C10—N2	−13.5 (6)	O4—C29—C30—N4	5.2 (6)
O3—C6—C7—C2	177.4 (3)	O6—C26—C27—C22	−178.1 (3)
O3—C6—C7—C8	2.7 (7)	O6—C26—C27—C28	−0.7 (7)
O3—C14—C15—C16	−141.2 (4)	O6—C34—C35—C36	−34.8 (6)
O3—C14—C15—C20	38.2 (6)	O6—C34—C35—C40	145.0 (4)
N1—C1—C8—C7	0.2 (5)	N3—C21—C28—C27	−0.4 (5)
N1—C1—C8—C9	−173.3 (4)	N3—C21—C28—C29	174.1 (4)
N1—C2—C3—C4	178.1 (5)	N3—C22—C23—C24	179.5 (5)
N1—C2—C7—C6	−178.0 (3)	N3—C22—C27—C26	179.2 (4)
N1—C2—C7—C8	−1.8 (4)	N3—C22—C27—C28	1.0 (4)
C1—N1—C2—C3	−176.3 (5)	C21—N3—C22—C23	178.0 (5)
C1—N1—C2—C7	2.0 (5)	C21—N3—C22—C27	−1.3 (5)
C1—C8—C9—O1	147.7 (5)	C21—C28—C29—O4	−153.3 (5)
C1—C8—C9—C10	−30.9 (6)	C21—C28—C29—C30	26.2 (6)
C2—N1—C1—C8	−1.4 (5)	C22—N3—C21—C28	1.1 (5)
C2—C3—C4—C5	0.0 (8)	C22—C23—C24—C25	1.6 (8)
C2—C7—C8—C1	1.0 (4)	C22—C27—C28—C21	−0.4 (4)
C2—C7—C8—C9	173.9 (4)	C22—C27—C28—C29	−174.3 (4)
C3—C2—C7—C6	0.4 (6)	C23—C22—C27—C26	−0.1 (6)
C3—C2—C7—C8	176.6 (4)	C23—C22—C27—C28	−178.3 (4)
C3—C4—C5—C6	−0.6 (8)	C23—C24—C25—C26	−0.6 (8)
C4—C5—C6—O3	−177.2 (4)	C24—C25—C26—O6	178.4 (4)
C4—C5—C6—C7	1.1 (6)	C24—C25—C26—C27	−0.9 (7)
C5—C6—C7—C2	−1.0 (5)	C25—C26—C27—C22	1.2 (6)
C5—C6—C7—C8	−175.7 (4)	C25—C26—C27—C28	178.6 (4)
C6—O3—C14—C15	179.6 (4)	C26—O6—C34—C35	−178.2 (4)
C6—C7—C8—C1	176.1 (4)	C26—C27—C28—C21	−178.0 (5)
C6—C7—C8—C9	−11.0 (8)	C26—C27—C28—C29	8.1 (8)
C7—C2—C3—C4	0.1 (7)	C27—C22—C23—C24	−1.3 (7)
C7—C8—C9—O1	−24.1 (7)	C27—C28—C29—O4	19.7 (7)
C7—C8—C9—C10	157.3 (4)	C27—C28—C29—C30	−160.8 (4)
C8—C9—C10—O2	−16.4 (6)	C28—C29—C30—O5	8.8 (6)
C8—C9—C10—N2	165.2 (4)	C28—C29—C30—N4	−174.3 (4)
C10—N2—C11—C12	−146.4 (5)	C30—N4—C31—C32	−81.7 (7)
C10—N2—C11—C13	90.0 (6)	C30—N4—C31—C33	153.3 (5)
C11—N2—C10—O2	3.5 (7)	C31—N4—C30—O5	−4.7 (8)
C11—N2—C10—C9	−178.1 (4)	C31—N4—C30—C29	178.5 (4)
C14—O3—C6—C5	−18.0 (6)	C34—O6—C26—C25	10.6 (6)
C14—O3—C6—C7	163.8 (4)	C34—O6—C26—C27	−170.1 (4)
C14—C15—C16—C17	−178.9 (4)	C34—C35—C36—C37	−179.0 (5)
C14—C15—C20—C19	178.4 (5)	C34—C35—C40—C39	179.4 (4)
C15—C16—C17—C18	0.1 (8)	C35—C36—C37—C38	−0.5 (10)
C16—C15—C20—C19	−2.1 (8)	C36—C35—C40—C39	−0.8 (7)
C16—C17—C18—C19	−1.3 (9)	C36—C37—C38—C39	−0.6 (10)
C17—C18—C19—C20	0.7 (10)	C37—C38—C39—C40	1.0 (9)
C18—C19—C20—C15	1.0 (10)	C38—C39—C40—C35	−0.3 (8)
C20—C15—C16—C17	1.6 (7)	C40—C35—C36—C37	1.2 (8)

### 2-[4-(Benzyloxy)-1H-indol-3-yl]-2-oxo-N-(propan-2-yl)acetamide (2) Hydrogen-bond geometry (Å, º)

**Table:**

*D*—H···*A*	*D*—H	H···*A*	*D*···*A*	*D*—H···*A*
N1—H1···O5^i^	0.86	1.98	2.810 (5)	164
N3—H3*A*···O2	0.87 (2)	1.99 (3)	2.801 (5)	154 (5)

Symmetry code: (i) *x*+1/2, −*y*+1/2, *z*.

### {2-[4-(Benzyloxy)-1H-indol-3-yl]ethyl}(propan-2-yl)azanium chloride (3a) Crystal data

**Table:**

C_20_H_25_N_2_O^+^·Cl^−^	*Z* = 4
*M**_r_* = 344.87	*F*(000) = 736
Triclinic, *P*1	*D*_x_ = 1.188 Mg m^−^^3^
*a* = 10.1895 (9) Å	Mo *K*α radiation, λ = 0.71073 Å
*b* = 10.9117 (7) Å	Cell parameters from 9870 reflections
*c* = 17.6887 (14) Å	θ = 2.8–25.6°
α = 86.798 (3)°	µ = 0.21 mm^−^^1^
β = 79.340 (3)°	*T* = 300 K
γ = 87.587 (3)°	Blcok, colourless
*V* = 1928.7 (3) Å^3^	0.30 × 0.27 × 0.20 mm

### {2-[4-(Benzyloxy)-1H-indol-3-yl]ethyl}(propan-2-yl)azanium chloride (3a) Data collection

**Table:**

Bruker D8 Venture CMOS diffractometer	5589 reflections with *I* > 2σ(*I*)
φ and ω scans	*R*_int_ = 0.033
Absorption correction: multi-scan (SADABS; Bruker, 2021)	θ_max_ = 25.8°, θ_min_ = 2.7°
*T*_min_ = 0.658, *T*_max_ = 0.745	*h* = −12→12
42151 measured reflections	*k* = −13→13
7362 independent reflections	*l* = −21→21

### {2-[4-(Benzyloxy)-1H-indol-3-yl]ethyl}(propan-2-yl)azanium chloride (3a) Refinement

**Table:**

Refinement on *F*^2^	6 restraints
Least-squares matrix: full	Hydrogen site location: mixed
*R*[*F*^2^ > 2σ(*F*^2^)] = 0.041	H atoms treated by a mixture of independent and constrained refinement
*wR*(*F*^2^) = 0.113	*w* = 1/[σ^2^(*F*_o_^2^) + (0.0453*P*)^2^ + 0.7204*P*] where *P* = (*F*_o_^2^ + 2*F*_c_^2^)/3
*S* = 1.01	(Δ/σ)_max_ < 0.001
7362 reflections	Δρ_max_ = 0.26 e Å^−^^3^
461 parameters	Δρ_min_ = −0.29 e Å^−^^3^

### {2-[4-(Benzyloxy)-1H-indol-3-yl]ethyl}(propan-2-yl)azanium chloride (3a) Special details

**Table:**

Geometry. All esds (except the esd in the dihedral angle between two l.s. planes) are estimated using the full covariance matrix. The cell esds are taken into account individually in the estimation of esds in distances, angles and torsion angles; correlations between esds in cell parameters are only used when they are defined by crystal symmetry. An approximate (isotropic) treatment of cell esds is used for estimating esds involving l.s. planes.

### {2-[4-(Benzyloxy)-1H-indol-3-yl]ethyl}(propan-2-yl)azanium chloride (3a) Fractional atomic coordinates and isotropic or equivalent isotropic displacement parameters (Å^2^)

**Table:**

	*x*	*y*	*z*	*U*_iso_*/*U*_eq_	
Cl1	0.92323 (5)	0.67300 (4)	0.44633 (3)	0.05824 (14)	
Cl2	0.34634 (5)	0.14033 (4)	−0.04745 (3)	0.05806 (14)	
O1	0.96246 (14)	0.75588 (11)	0.81683 (7)	0.0543 (3)	
O2	0.58220 (15)	0.24805 (12)	0.28702 (8)	0.0599 (4)	
N1	1.00390 (18)	1.05554 (15)	0.62139 (10)	0.0569 (4)	
N2	0.88071 (16)	0.54312 (14)	0.61023 (9)	0.0472 (4)	
N3	0.55205 (18)	0.60081 (15)	0.13621 (10)	0.0571 (4)	
N4	0.44895 (16)	0.07170 (13)	0.10585 (8)	0.0447 (3)	
C1	0.9363 (2)	0.95917 (17)	0.60396 (11)	0.0522 (4)	
H1A	0.902311	0.955148	0.558905	0.063*	
C2	1.03828 (19)	1.03071 (16)	0.69185 (11)	0.0493 (4)	
C3	1.1079 (2)	1.10215 (18)	0.73283 (13)	0.0618 (5)	
H3	1.138537	1.178820	0.713479	0.074*	
C4	1.1292 (2)	1.05469 (19)	0.80252 (13)	0.0685 (6)	
H4	1.176174	1.099975	0.831038	0.082*	
C5	1.0825 (2)	0.93968 (19)	0.83272 (12)	0.0622 (5)	
H5	1.098189	0.910655	0.880799	0.075*	
C6	1.01364 (19)	0.86924 (16)	0.79188 (10)	0.0488 (4)	
C7	0.99032 (17)	0.91381 (15)	0.71935 (10)	0.0422 (4)	
C8	0.92513 (17)	0.86917 (15)	0.66134 (10)	0.0429 (4)	
C9	0.85418 (17)	0.75285 (16)	0.65954 (11)	0.0478 (4)	
H9A	0.801952	0.761737	0.618827	0.057*	
H9B	0.792351	0.740893	0.707906	0.057*	
C10	0.94459 (19)	0.63883 (16)	0.64680 (11)	0.0504 (4)	
H10A	1.028450	0.660588	0.614039	0.060*	
H10B	0.963891	0.606101	0.695828	0.060*	
C11	0.7499 (2)	0.4955 (2)	0.65233 (14)	0.0660 (6)	
H11	0.692227	0.565425	0.671627	0.079*	
C12	0.6860 (3)	0.4335 (3)	0.5953 (2)	0.1251 (13)	
H12A	0.600112	0.405005	0.620140	0.188*	
H12B	0.741760	0.365091	0.575636	0.188*	
H12C	0.675264	0.490939	0.553517	0.188*	
C13	0.7723 (3)	0.4133 (3)	0.71931 (19)	0.1196 (12)	
H13A	0.688711	0.380818	0.745018	0.179*	
H13B	0.809615	0.459136	0.754522	0.179*	
H13C	0.832962	0.346907	0.701486	0.179*	
C14	0.9880 (3)	0.70915 (19)	0.89042 (12)	0.0694 (6)	
H14A	0.941757	0.760771	0.930509	0.083*	
H14B	1.083012	0.710211	0.890900	0.083*	
C15	0.9408 (2)	0.58056 (17)	0.90557 (11)	0.0529 (5)	
C16	1.0015 (3)	0.4873 (2)	0.86211 (13)	0.0793 (7)	
H16	1.069007	0.504242	0.820162	0.095*	
C17	0.9628 (4)	0.3679 (2)	0.88032 (17)	0.0979 (10)	
H17	1.003586	0.304756	0.850290	0.117*	
C18	0.8641 (3)	0.3422 (2)	0.94272 (17)	0.0867 (8)	
H18	0.838464	0.261697	0.955274	0.104*	
C19	0.8042 (2)	0.4345 (2)	0.98584 (15)	0.0744 (7)	
H19	0.737486	0.417467	1.028176	0.089*	
C20	0.8420 (2)	0.55345 (19)	0.96718 (12)	0.0604 (5)	
H20	0.799728	0.616432	0.996864	0.072*	
C21	0.4874 (2)	0.50805 (17)	0.11145 (11)	0.0540 (5)	
H21	0.443699	0.515233	0.069597	0.065*	
C22	0.60418 (18)	0.55794 (16)	0.19888 (11)	0.0492 (4)	
C23	0.6753 (2)	0.6196 (2)	0.24423 (13)	0.0636 (5)	
H23	0.695515	0.701766	0.233628	0.076*	
C24	0.7138 (2)	0.5547 (2)	0.30457 (14)	0.0701 (6)	
H24	0.760723	0.593864	0.336106	0.084*	
C25	0.6851 (2)	0.4307 (2)	0.32078 (12)	0.0630 (5)	
H25	0.713598	0.389018	0.362452	0.076*	
C26	0.61511 (19)	0.36954 (17)	0.27569 (10)	0.0492 (4)	
C27	0.57152 (17)	0.43356 (15)	0.21327 (9)	0.0423 (4)	
C28	0.49560 (18)	0.40345 (15)	0.15642 (9)	0.0438 (4)	
C29	0.42841 (18)	0.28675 (16)	0.14869 (10)	0.0483 (4)	
H29A	0.367867	0.302283	0.112632	0.058*	
H29B	0.375132	0.262903	0.198241	0.058*	
C30	0.52222 (18)	0.18076 (16)	0.12175 (10)	0.0469 (4)	
H30A	0.582922	0.206845	0.075240	0.056*	
H30B	0.575094	0.157353	0.160871	0.056*	
C31	0.3427 (2)	0.02117 (18)	0.16887 (11)	0.0548 (5)	
H31	0.275339	0.086446	0.183618	0.066*	
C32	0.4012 (3)	−0.0205 (2)	0.23822 (13)	0.0850 (8)	
H32A	0.436550	0.048546	0.258190	0.128*	
H32B	0.471660	−0.080659	0.223843	0.128*	
H32C	0.332963	−0.056095	0.276988	0.128*	
C33	0.2771 (2)	−0.0812 (2)	0.13661 (15)	0.0756 (7)	
H33A	0.204093	−0.111016	0.174616	0.113*	
H33B	0.341352	−0.147018	0.123327	0.113*	
H33C	0.244225	−0.050541	0.091469	0.113*	
C34	0.6554 (2)	0.1705 (2)	0.33397 (12)	0.0656 (6)	
H34A	0.747301	0.196068	0.324815	0.079*	
H34B	0.655949	0.086874	0.318031	0.079*	
C35	0.60064 (19)	0.17215 (17)	0.41881 (11)	0.0516 (4)	
C36	0.4761 (2)	0.2165 (3)	0.44842 (15)	0.0865 (8)	
H36	0.422403	0.251246	0.415227	0.104*	
C37	0.4278 (3)	0.2110 (3)	0.52678 (16)	0.0948 (9)	
H37	0.342522	0.242462	0.545606	0.114*	
C38	0.5026 (3)	0.1608 (2)	0.57590 (14)	0.0775 (7)	
H38	0.469508	0.155762	0.628582	0.093*	
C39	0.6263 (3)	0.1178 (3)	0.54767 (14)	0.0979 (9)	
H39	0.679341	0.083373	0.581328	0.117*	
C40	0.6759 (3)	0.1238 (3)	0.46979 (13)	0.0837 (8)	
H40	0.762384	0.094324	0.451731	0.100*	
H1	1.015 (2)	1.1243 (12)	0.5942 (11)	0.067 (6)*	
H2A	0.9443 (16)	0.4778 (14)	0.6006 (11)	0.063 (6)*	
H2B	0.868 (2)	0.5789 (17)	0.5622 (7)	0.064 (6)*	
H3A	0.567 (2)	0.6707 (13)	0.1113 (12)	0.082 (8)*	
H4A	0.5134 (15)	0.0084 (13)	0.0920 (10)	0.053 (5)*	
H4B	0.4086 (19)	0.0901 (18)	0.0626 (8)	0.061 (6)*	

### {2-[4-(Benzyloxy)-1H-indol-3-yl]ethyl}(propan-2-yl)azanium chloride (3a) Atomic displacement parameters (Å^2^)

**Table:**

	*U*^11^	*U*^22^	*U*^33^	*U*^12^	*U*^13^	*U*^23^
Cl1	0.0705 (3)	0.0464 (3)	0.0552 (3)	0.0067 (2)	−0.0081 (2)	0.0021 (2)
Cl2	0.0782 (4)	0.0465 (3)	0.0504 (3)	0.0089 (2)	−0.0167 (2)	−0.00195 (19)
O1	0.0714 (9)	0.0488 (7)	0.0461 (7)	−0.0095 (6)	−0.0207 (6)	0.0075 (5)
O2	0.0800 (10)	0.0513 (8)	0.0553 (8)	−0.0016 (7)	−0.0317 (7)	0.0025 (6)
N1	0.0722 (11)	0.0430 (9)	0.0551 (10)	−0.0021 (8)	−0.0136 (8)	0.0085 (7)
N2	0.0496 (9)	0.0416 (8)	0.0488 (9)	−0.0019 (7)	−0.0038 (7)	−0.0059 (7)
N3	0.0707 (11)	0.0428 (9)	0.0561 (10)	−0.0033 (8)	−0.0077 (8)	0.0020 (7)
N4	0.0547 (9)	0.0424 (8)	0.0372 (8)	−0.0007 (7)	−0.0072 (7)	−0.0075 (6)
C1	0.0586 (12)	0.0526 (11)	0.0454 (10)	0.0041 (9)	−0.0110 (9)	−0.0011 (8)
C2	0.0543 (11)	0.0406 (9)	0.0521 (10)	0.0001 (8)	−0.0082 (8)	−0.0017 (8)
C3	0.0728 (14)	0.0436 (11)	0.0716 (13)	−0.0110 (9)	−0.0179 (11)	−0.0015 (9)
C4	0.0830 (16)	0.0544 (12)	0.0760 (15)	−0.0141 (11)	−0.0301 (12)	−0.0107 (10)
C5	0.0782 (15)	0.0588 (12)	0.0558 (12)	−0.0073 (10)	−0.0279 (11)	−0.0006 (9)
C6	0.0560 (11)	0.0430 (10)	0.0483 (10)	−0.0014 (8)	−0.0119 (8)	−0.0013 (8)
C7	0.0439 (9)	0.0380 (9)	0.0443 (9)	0.0011 (7)	−0.0066 (7)	−0.0037 (7)
C8	0.0424 (9)	0.0435 (9)	0.0421 (9)	0.0039 (7)	−0.0060 (7)	−0.0053 (7)
C9	0.0411 (10)	0.0505 (10)	0.0506 (10)	0.0004 (8)	−0.0034 (8)	−0.0118 (8)
C10	0.0492 (11)	0.0479 (10)	0.0556 (11)	−0.0003 (8)	−0.0127 (8)	−0.0059 (8)
C11	0.0536 (12)	0.0550 (12)	0.0834 (15)	−0.0090 (9)	0.0065 (11)	−0.0087 (10)
C12	0.085 (2)	0.125 (3)	0.171 (3)	−0.0436 (19)	−0.014 (2)	−0.052 (2)
C13	0.101 (2)	0.113 (2)	0.116 (2)	0.0037 (18)	0.0356 (19)	0.049 (2)
C14	0.0998 (18)	0.0593 (13)	0.0554 (12)	−0.0121 (12)	−0.0327 (12)	0.0105 (10)
C15	0.0661 (13)	0.0496 (11)	0.0453 (10)	0.0032 (9)	−0.0192 (9)	0.0041 (8)
C16	0.103 (2)	0.0744 (16)	0.0540 (13)	0.0122 (13)	−0.0007 (12)	−0.0045 (11)
C17	0.158 (3)	0.0610 (16)	0.0798 (18)	0.0265 (17)	−0.0355 (19)	−0.0222 (13)
C18	0.125 (2)	0.0573 (15)	0.0916 (19)	−0.0223 (15)	−0.0574 (18)	0.0139 (13)
C19	0.0616 (14)	0.0821 (17)	0.0806 (16)	−0.0122 (12)	−0.0211 (12)	0.0216 (13)
C20	0.0604 (13)	0.0627 (13)	0.0576 (12)	0.0125 (10)	−0.0146 (10)	0.0020 (9)
C21	0.0631 (12)	0.0541 (11)	0.0453 (10)	0.0054 (9)	−0.0124 (9)	−0.0035 (8)
C22	0.0472 (10)	0.0475 (10)	0.0503 (10)	−0.0033 (8)	−0.0007 (8)	−0.0071 (8)
C23	0.0623 (13)	0.0569 (12)	0.0718 (14)	−0.0165 (10)	−0.0057 (11)	−0.0160 (10)
C24	0.0637 (14)	0.0817 (16)	0.0701 (14)	−0.0203 (12)	−0.0157 (11)	−0.0232 (12)
C25	0.0601 (13)	0.0785 (15)	0.0554 (12)	−0.0076 (11)	−0.0213 (10)	−0.0064 (10)
C26	0.0492 (11)	0.0525 (11)	0.0468 (10)	−0.0026 (8)	−0.0100 (8)	−0.0061 (8)
C27	0.0417 (9)	0.0441 (9)	0.0404 (9)	−0.0010 (7)	−0.0043 (7)	−0.0080 (7)
C28	0.0475 (10)	0.0447 (9)	0.0389 (9)	0.0024 (7)	−0.0060 (7)	−0.0073 (7)
C29	0.0484 (10)	0.0508 (10)	0.0467 (10)	0.0001 (8)	−0.0091 (8)	−0.0108 (8)
C30	0.0484 (10)	0.0483 (10)	0.0436 (9)	−0.0032 (8)	−0.0046 (8)	−0.0105 (7)
C31	0.0597 (12)	0.0527 (11)	0.0489 (10)	−0.0084 (9)	0.0001 (9)	−0.0048 (8)
C32	0.108 (2)	0.0951 (18)	0.0491 (12)	−0.0190 (15)	−0.0087 (13)	0.0137 (12)
C33	0.0767 (16)	0.0639 (14)	0.0851 (16)	−0.0214 (12)	−0.0064 (13)	−0.0075 (12)
C34	0.0764 (15)	0.0659 (13)	0.0562 (12)	0.0195 (11)	−0.0217 (11)	−0.0027 (10)
C35	0.0540 (11)	0.0476 (10)	0.0555 (11)	0.0055 (8)	−0.0187 (9)	−0.0005 (8)
C36	0.0594 (14)	0.128 (2)	0.0729 (16)	0.0244 (14)	−0.0234 (12)	0.0059 (15)
C37	0.0599 (15)	0.134 (3)	0.0820 (18)	0.0177 (15)	0.0019 (13)	0.0025 (17)
C38	0.0893 (18)	0.0795 (16)	0.0592 (13)	0.0041 (13)	−0.0058 (13)	0.0061 (11)
C39	0.107 (2)	0.128 (2)	0.0575 (14)	0.0461 (19)	−0.0248 (14)	0.0058 (14)
C40	0.0757 (16)	0.112 (2)	0.0617 (14)	0.0441 (14)	−0.0193 (12)	−0.0026 (13)

### {2-[4-(Benzyloxy)-1H-indol-3-yl]ethyl}(propan-2-yl)azanium chloride (3a) Geometric parameters (Å, º)

**Table:**

O1—C6	1.380 (2)	C16—H16	0.9300
O1—C14	1.436 (2)	C16—C17	1.380 (4)
O2—C26	1.376 (2)	C17—H17	0.9300
O2—C34	1.435 (2)	C17—C18	1.374 (4)
N1—C1	1.360 (3)	C18—H18	0.9300
N1—C2	1.366 (2)	C18—C19	1.353 (4)
N1—H1	0.868 (9)	C19—H19	0.9300
N2—C10	1.490 (2)	C19—C20	1.374 (3)
N2—C11	1.500 (2)	C20—H20	0.9300
N2—H2A	0.944 (9)	C21—H21	0.9300
N2—H2B	0.945 (9)	C21—C28	1.363 (2)
N3—C21	1.365 (3)	C22—C23	1.392 (3)
N3—C22	1.367 (2)	C22—C27	1.409 (2)
N3—H3A	0.863 (10)	C23—H23	0.9300
N4—C30	1.498 (2)	C23—C24	1.357 (3)
N4—C31	1.501 (2)	C24—H24	0.9300
N4—H4A	0.943 (9)	C24—C25	1.399 (3)
N4—H4B	0.941 (9)	C25—H25	0.9300
C1—H1A	0.9300	C25—C26	1.378 (3)
C1—C8	1.365 (2)	C26—C27	1.405 (2)
C2—C3	1.392 (3)	C27—C28	1.437 (2)
C2—C7	1.413 (2)	C28—C29	1.495 (2)
C3—H3	0.9300	C29—H29A	0.9700
C3—C4	1.361 (3)	C29—H29B	0.9700
C4—H4	0.9300	C29—C30	1.509 (2)
C4—C5	1.403 (3)	C30—H30A	0.9700
C5—H5	0.9300	C30—H30B	0.9700
C5—C6	1.378 (3)	C31—H31	0.9800
C6—C7	1.405 (2)	C31—C32	1.501 (3)
C7—C8	1.438 (2)	C31—C33	1.516 (3)
C8—C9	1.492 (2)	C32—H32A	0.9600
C9—H9A	0.9700	C32—H32B	0.9600
C9—H9B	0.9700	C32—H32C	0.9600
C9—C10	1.518 (2)	C33—H33A	0.9600
C10—H10A	0.9700	C33—H33B	0.9600
C10—H10B	0.9700	C33—H33C	0.9600
C11—H11	0.9800	C34—H34A	0.9700
C11—C12	1.503 (4)	C34—H34B	0.9700
C11—C13	1.492 (4)	C34—C35	1.502 (3)
C12—H12A	0.9600	C35—C36	1.360 (3)
C12—H12B	0.9600	C35—C40	1.360 (3)
C12—H12C	0.9600	C36—H36	0.9300
C13—H13A	0.9600	C36—C37	1.381 (4)
C13—H13B	0.9600	C37—H37	0.9300
C13—H13C	0.9600	C37—C38	1.339 (3)
C14—H14A	0.9700	C38—H38	0.9300
C14—H14B	0.9700	C38—C39	1.342 (4)
C14—C15	1.497 (3)	C39—H39	0.9300
C15—C16	1.367 (3)	C39—C40	1.376 (3)
C15—C20	1.368 (3)	C40—H40	0.9300
			
C6—O1—C14	115.71 (14)	C18—C17—H17	119.9
C26—O2—C34	117.54 (16)	C17—C18—H18	120.2
C1—N1—C2	108.70 (15)	C19—C18—C17	119.7 (2)
C1—N1—H1	124.7 (15)	C19—C18—H18	120.2
C2—N1—H1	126.2 (15)	C18—C19—H19	119.9
C10—N2—C11	117.90 (15)	C18—C19—C20	120.1 (2)
C10—N2—H2A	106.6 (13)	C20—C19—H19	119.9
C10—N2—H2B	105.5 (13)	C15—C20—C19	120.9 (2)
C11—N2—H2A	110.2 (13)	C15—C20—H20	119.5
C11—N2—H2B	108.4 (13)	C19—C20—H20	119.5
H2A—N2—H2B	107.8 (18)	N3—C21—H21	124.6
C21—N3—C22	108.79 (15)	C28—C21—N3	110.75 (17)
C21—N3—H3A	123.8 (17)	C28—C21—H21	124.6
C22—N3—H3A	126.8 (17)	N3—C22—C23	129.43 (18)
C30—N4—C31	118.11 (13)	N3—C22—C27	107.60 (16)
C30—N4—H4A	107.1 (12)	C23—C22—C27	122.96 (19)
C30—N4—H4B	109.6 (12)	C22—C23—H23	121.4
C31—N4—H4A	108.1 (12)	C24—C23—C22	117.2 (2)
C31—N4—H4B	107.0 (13)	C24—C23—H23	121.4
H4A—N4—H4B	106.4 (17)	C23—C24—H24	119.0
N1—C1—H1A	124.5	C23—C24—C25	122.00 (19)
N1—C1—C8	111.04 (17)	C25—C24—H24	119.0
C8—C1—H1A	124.5	C24—C25—H25	119.6
N1—C2—C3	129.08 (18)	C26—C25—C24	120.8 (2)
N1—C2—C7	107.80 (16)	C26—C25—H25	119.6
C3—C2—C7	123.13 (18)	O2—C26—C25	124.99 (18)
C2—C3—H3	121.5	O2—C26—C27	115.83 (15)
C4—C3—C2	116.96 (18)	C25—C26—C27	119.19 (18)
C4—C3—H3	121.5	C22—C27—C28	107.04 (15)
C3—C4—H4	118.9	C26—C27—C22	117.85 (16)
C3—C4—C5	122.12 (19)	C26—C27—C28	135.10 (16)
C5—C4—H4	118.9	C21—C28—C27	105.81 (16)
C4—C5—H5	119.7	C21—C28—C29	124.87 (17)
C6—C5—C4	120.69 (19)	C27—C28—C29	129.18 (16)
C6—C5—H5	119.7	C28—C29—H29A	108.6
O1—C6—C7	115.58 (15)	C28—C29—H29B	108.6
C5—C6—O1	125.13 (17)	C28—C29—C30	114.73 (15)
C5—C6—C7	119.28 (17)	H29A—C29—H29B	107.6
C2—C7—C8	106.75 (15)	C30—C29—H29A	108.6
C6—C7—C2	117.81 (16)	C30—C29—H29B	108.6
C6—C7—C8	135.44 (16)	N4—C30—C29	112.12 (15)
C1—C8—C7	105.72 (16)	N4—C30—H30A	109.2
C1—C8—C9	123.63 (17)	N4—C30—H30B	109.2
C7—C8—C9	130.62 (16)	C29—C30—H30A	109.2
C8—C9—H9A	108.6	C29—C30—H30B	109.2
C8—C9—H9B	108.6	H30A—C30—H30B	107.9
C8—C9—C10	114.85 (15)	N4—C31—H31	108.5
H9A—C9—H9B	107.5	N4—C31—C33	107.79 (16)
C10—C9—H9A	108.6	C32—C31—N4	110.56 (18)
C10—C9—H9B	108.6	C32—C31—H31	108.5
N2—C10—C9	111.09 (15)	C32—C31—C33	112.83 (19)
N2—C10—H10A	109.4	C33—C31—H31	108.5
N2—C10—H10B	109.4	C31—C32—H32A	109.5
C9—C10—H10A	109.4	C31—C32—H32B	109.5
C9—C10—H10B	109.4	C31—C32—H32C	109.5
H10A—C10—H10B	108.0	H32A—C32—H32B	109.5
N2—C11—H11	108.6	H32A—C32—H32C	109.5
N2—C11—C12	107.6 (2)	H32B—C32—H32C	109.5
C12—C11—H11	108.6	C31—C33—H33A	109.5
C13—C11—N2	109.8 (2)	C31—C33—H33B	109.5
C13—C11—H11	108.6	C31—C33—H33C	109.5
C13—C11—C12	113.6 (3)	H33A—C33—H33B	109.5
C11—C12—H12A	109.5	H33A—C33—H33C	109.5
C11—C12—H12B	109.5	H33B—C33—H33C	109.5
C11—C12—H12C	109.5	O2—C34—H34A	108.7
H12A—C12—H12B	109.5	O2—C34—H34B	108.7
H12A—C12—H12C	109.5	O2—C34—C35	114.29 (17)
H12B—C12—H12C	109.5	H34A—C34—H34B	107.6
C11—C13—H13A	109.5	C35—C34—H34A	108.7
C11—C13—H13B	109.5	C35—C34—H34B	108.7
C11—C13—H13C	109.5	C36—C35—C34	123.43 (19)
H13A—C13—H13B	109.5	C36—C35—C40	117.1 (2)
H13A—C13—H13C	109.5	C40—C35—C34	119.48 (19)
H13B—C13—H13C	109.5	C35—C36—H36	119.3
O1—C14—H14A	109.7	C35—C36—C37	121.4 (2)
O1—C14—H14B	109.8	C37—C36—H36	119.3
O1—C14—C15	109.60 (16)	C36—C37—H37	119.7
H14A—C14—H14B	108.2	C38—C37—C36	120.6 (2)
C15—C14—H14A	109.7	C38—C37—H37	119.7
C15—C14—H14B	109.8	C37—C38—H38	120.6
C16—C15—C14	121.0 (2)	C37—C38—C39	118.7 (2)
C16—C15—C20	119.0 (2)	C39—C38—H38	120.6
C20—C15—C14	119.89 (19)	C38—C39—H39	119.4
C15—C16—H16	119.9	C38—C39—C40	121.1 (2)
C15—C16—C17	120.2 (2)	C40—C39—H39	119.4
C17—C16—H16	119.9	C35—C40—C39	121.0 (2)
C16—C17—H17	119.9	C35—C40—H40	119.5
C18—C17—C16	120.1 (2)	C39—C40—H40	119.5
			
O1—C6—C7—C2	178.71 (16)	C14—O1—C6—C7	179.02 (18)
O1—C6—C7—C8	−0.9 (3)	C14—C15—C16—C17	175.4 (2)
O1—C14—C15—C16	66.8 (3)	C14—C15—C20—C19	−174.8 (2)
O1—C14—C15—C20	−118.0 (2)	C15—C16—C17—C18	−0.7 (4)
O2—C26—C27—C22	−179.31 (15)	C16—C15—C20—C19	0.4 (3)
O2—C26—C27—C28	1.4 (3)	C16—C17—C18—C19	0.5 (4)
O2—C34—C35—C36	15.9 (3)	C17—C18—C19—C20	0.1 (4)
O2—C34—C35—C40	−166.3 (2)	C18—C19—C20—C15	−0.6 (3)
N1—C1—C8—C7	0.4 (2)	C20—C15—C16—C17	0.2 (4)
N1—C1—C8—C9	178.92 (16)	C21—N3—C22—C23	−178.5 (2)
N1—C2—C3—C4	−179.8 (2)	C21—N3—C22—C27	0.4 (2)
N1—C2—C7—C6	−179.56 (16)	C21—C28—C29—C30	112.7 (2)
N1—C2—C7—C8	0.1 (2)	C22—N3—C21—C28	−0.2 (2)
N3—C21—C28—C27	−0.2 (2)	C22—C23—C24—C25	0.6 (3)
N3—C21—C28—C29	175.82 (16)	C22—C27—C28—C21	0.45 (19)
N3—C22—C23—C24	178.9 (2)	C22—C27—C28—C29	−175.33 (17)
N3—C22—C27—C26	−179.99 (16)	C23—C22—C27—C26	−1.0 (3)
N3—C22—C27—C28	−0.54 (19)	C23—C22—C27—C28	178.44 (17)
C1—N1—C2—C3	179.9 (2)	C23—C24—C25—C26	−0.4 (3)
C1—N1—C2—C7	0.1 (2)	C24—C25—C26—O2	−179.99 (19)
C1—C8—C9—C10	108.3 (2)	C24—C25—C26—C27	−0.5 (3)
C2—N1—C1—C8	−0.3 (2)	C25—C26—C27—C22	1.2 (3)
C2—C3—C4—C5	−0.6 (3)	C25—C26—C27—C28	−178.05 (19)
C2—C7—C8—C1	−0.32 (19)	C26—O2—C34—C35	85.8 (2)
C2—C7—C8—C9	−178.69 (17)	C26—C27—C28—C21	179.8 (2)
C3—C2—C7—C6	0.6 (3)	C26—C27—C28—C29	4.0 (3)
C3—C2—C7—C8	−179.69 (18)	C27—C22—C23—C24	0.1 (3)
C3—C4—C5—C6	0.7 (4)	C27—C28—C29—C30	−72.3 (2)
C4—C5—C6—O1	−179.2 (2)	C28—C29—C30—N4	−172.94 (14)
C4—C5—C6—C7	0.0 (3)	C30—N4—C31—C32	−60.6 (2)
C5—C6—C7—C2	−0.6 (3)	C30—N4—C31—C33	175.65 (17)
C5—C6—C7—C8	179.9 (2)	C31—N4—C30—C29	−54.9 (2)
C6—O1—C14—C15	−173.52 (17)	C34—O2—C26—C25	−18.4 (3)
C6—C7—C8—C1	179.3 (2)	C34—O2—C26—C27	162.13 (16)
C6—C7—C8—C9	0.9 (3)	C34—C35—C36—C37	177.0 (3)
C7—C2—C3—C4	0.0 (3)	C34—C35—C40—C39	−176.5 (3)
C7—C8—C9—C10	−73.5 (2)	C35—C36—C37—C38	−0.4 (5)
C8—C9—C10—N2	−153.55 (15)	C36—C35—C40—C39	1.5 (4)
C10—N2—C11—C12	162.3 (2)	C36—C37—C38—C39	1.1 (5)
C10—N2—C11—C13	−73.6 (3)	C37—C38—C39—C40	−0.5 (5)
C11—N2—C10—C9	−59.7 (2)	C38—C39—C40—C35	−0.8 (5)
C14—O1—C6—C5	−1.8 (3)	C40—C35—C36—C37	−0.9 (4)

### {2-[4-(Benzyloxy)-1H-indol-3-yl]ethyl}(propan-2-yl)azanium chloride (3a) Hydrogen-bond geometry (Å, º)

**Table:**

*D*—H···*A*	*D*—H	H···*A*	*D*···*A*	*D*—H···*A*
N1—H1···Cl1^i^	0.87 (1)	2.36 (1)	3.1969 (17)	162 (2)
N2—H2*A*···Cl1^ii^	0.94 (1)	2.18 (1)	3.1114 (16)	167 (2)
N2—H2*B*···Cl1	0.95 (1)	2.23 (1)	3.1191 (16)	157 (2)
N3—H3*A*···Cl2^iii^	0.86 (1)	2.42 (1)	3.2657 (17)	168 (2)
N4—H4*A*···Cl2^iv^	0.94 (1)	2.20 (1)	3.1360 (16)	173 (2)
N4—H4*B*···Cl2	0.94 (1)	2.19 (1)	3.1247 (16)	171 (2)

Symmetry codes: (i) −*x*+2, −*y*+2, −*z*+1; (ii) −*x*+2, −*y*+1, −*z*+1; (iii) −*x*+1, −*y*+1, −*z*; (iv) −*x*+1, −*y*, −*z*.

### 3-[2-(Propan-2-ylamino)ethyl]-1H-indol-4-ol (4) Crystal data

**Table:**

C_13_H_18_N_2_O	*D*_x_ = 1.207 Mg m^−^^3^
*M**_r_* = 218.29	Mo *K*α radiation, λ = 0.71073 Å
Orthorhombic, *P**b**c**a*	Cell parameters from 9906 reflections
*a* = 8.4065 (5) Å	θ = 2.8–25.6°
*b* = 14.3944 (9) Å	µ = 0.08 mm^−^^1^
*c* = 19.8501 (10) Å	*T* = 300 K
*V* = 2402.0 (2) Å^3^	Block, colourless
*Z* = 8	0.33 × 0.25 × 0.20 mm
*F*(000) = 944	

### 3-[2-(Propan-2-ylamino)ethyl]-1H-indol-4-ol (4) Data collection

**Table:**

Bruker D8 Venture CMOS diffractometer	2121 reflections with *I* > 2σ(*I*)
φ and ω scans	*R*_int_ = 0.050
Absorption correction: multi-scan (SADABS; Bruker, 2021)	θ_max_ = 26.4°, θ_min_ = 2.8°
*T*_min_ = 0.715, *T*_max_ = 0.745	*h* = −10→10
61022 measured reflections	*k* = −18→18
2461 independent reflections	*l* = −24→24

### 3-[2-(Propan-2-ylamino)ethyl]-1H-indol-4-ol (4) Refinement

**Table:**

Refinement on *F*^2^	3 restraints
Least-squares matrix: full	Hydrogen site location: mixed
*R*[*F*^2^ > 2σ(*F*^2^)] = 0.045	H atoms treated by a mixture of independent and constrained refinement
*wR*(*F*^2^) = 0.116	*w* = 1/[σ^2^(*F*_o_^2^) + (0.0509*P*)^2^ + 0.7218*P*] where *P* = (*F*_o_^2^ + 2*F*_c_^2^)/3
*S* = 1.08	(Δ/σ)_max_ < 0.001
2461 reflections	Δρ_max_ = 0.18 e Å^−^^3^
159 parameters	Δρ_min_ = −0.15 e Å^−^^3^

### 3-[2-(Propan-2-ylamino)ethyl]-1H-indol-4-ol (4) Special details

**Table:**

Geometry. All esds (except the esd in the dihedral angle between two l.s. planes) are estimated using the full covariance matrix. The cell esds are taken into account individually in the estimation of esds in distances, angles and torsion angles; correlations between esds in cell parameters are only used when they are defined by crystal symmetry. An approximate (isotropic) treatment of cell esds is used for estimating esds involving l.s. planes.

### 3-[2-(Propan-2-ylamino)ethyl]-1H-indol-4-ol (4) Fractional atomic coordinates and isotropic or equivalent isotropic displacement parameters (Å^2^)

**Table:**

	*x*	*y*	*z*	*U*_iso_*/*U*_eq_	
O1	0.61118 (12)	0.61010 (6)	0.38334 (6)	0.0478 (3)	
N1	0.77344 (18)	0.91907 (9)	0.37395 (8)	0.0551 (4)	
N2	0.32230 (14)	0.67852 (8)	0.37951 (6)	0.0397 (3)	
C1	0.6437 (2)	0.90682 (11)	0.33371 (9)	0.0522 (4)	
H1B	0.595323	0.953994	0.309107	0.063*	
C2	0.81228 (17)	0.83572 (10)	0.40260 (7)	0.0406 (3)	
C3	0.93477 (18)	0.81377 (11)	0.44719 (8)	0.0496 (4)	
H3	1.006571	0.858574	0.461856	0.059*	
C4	0.94493 (19)	0.72347 (12)	0.46850 (8)	0.0496 (4)	
H4	1.025759	0.706585	0.498049	0.060*	
C5	0.83653 (18)	0.65601 (11)	0.44684 (8)	0.0439 (4)	
H5	0.846425	0.595429	0.462570	0.053*	
C6	0.71540 (16)	0.67743 (9)	0.40270 (7)	0.0350 (3)	
C7	0.70144 (15)	0.76948 (9)	0.37899 (6)	0.0332 (3)	
C8	0.59388 (17)	0.81690 (10)	0.33409 (7)	0.0400 (3)	
C9	0.45501 (19)	0.78061 (12)	0.29482 (8)	0.0496 (4)	
H9A	0.486563	0.723433	0.272682	0.060*	
H9B	0.428623	0.825257	0.259960	0.060*	
C10	0.30611 (18)	0.76164 (12)	0.33657 (9)	0.0516 (4)	
H10A	0.284311	0.815124	0.364830	0.062*	
H10B	0.216237	0.753324	0.306528	0.062*	
C11	0.21361 (17)	0.60121 (11)	0.36284 (8)	0.0458 (4)	
H11	0.105392	0.625769	0.358403	0.055*	
C12	0.2167 (2)	0.53158 (12)	0.41978 (10)	0.0643 (5)	
H12A	0.143267	0.482209	0.410295	0.096*	
H12B	0.186765	0.561735	0.461012	0.096*	
H12C	0.322036	0.506555	0.424211	0.096*	
C13	0.2623 (2)	0.55756 (15)	0.29654 (10)	0.0741 (6)	
H13A	0.190508	0.507916	0.285706	0.111*	
H13B	0.368466	0.533495	0.300308	0.111*	
H13C	0.258916	0.603620	0.261593	0.111*	
H2	0.304 (2)	0.6938 (12)	0.4226 (5)	0.054 (5)*	
H1A	0.822 (2)	0.9726 (9)	0.3790 (9)	0.071 (6)*	
H1	0.5026 (15)	0.6380 (13)	0.3800 (10)	0.077 (6)*	

### 3-[2-(Propan-2-ylamino)ethyl]-1H-indol-4-ol (4) Atomic displacement parameters (Å^2^)

**Table:**

	*U*^11^	*U*^22^	*U*^33^	*U*^12^	*U*^13^	*U*^23^
O1	0.0387 (6)	0.0262 (5)	0.0784 (8)	0.0011 (4)	−0.0065 (5)	−0.0003 (5)
N1	0.0535 (9)	0.0306 (7)	0.0813 (10)	−0.0091 (6)	0.0127 (7)	0.0033 (6)
N2	0.0369 (6)	0.0376 (6)	0.0445 (7)	−0.0001 (5)	−0.0022 (5)	−0.0035 (5)
C1	0.0510 (9)	0.0372 (8)	0.0684 (10)	0.0040 (7)	0.0142 (8)	0.0191 (7)
C2	0.0405 (7)	0.0342 (7)	0.0470 (8)	−0.0044 (6)	0.0132 (6)	−0.0054 (6)
C3	0.0405 (8)	0.0546 (9)	0.0537 (9)	−0.0082 (7)	0.0042 (7)	−0.0176 (7)
C4	0.0404 (8)	0.0643 (10)	0.0441 (8)	0.0062 (7)	−0.0037 (6)	−0.0081 (7)
C5	0.0419 (8)	0.0418 (8)	0.0479 (8)	0.0068 (6)	0.0002 (6)	0.0052 (6)
C6	0.0340 (7)	0.0289 (6)	0.0421 (7)	0.0026 (5)	0.0042 (6)	−0.0007 (5)
C7	0.0336 (7)	0.0304 (7)	0.0357 (6)	0.0009 (5)	0.0090 (5)	−0.0012 (5)
C8	0.0394 (7)	0.0367 (7)	0.0439 (7)	0.0035 (6)	0.0094 (6)	0.0085 (6)
C9	0.0478 (9)	0.0538 (9)	0.0473 (8)	0.0050 (7)	−0.0025 (7)	0.0134 (7)
C10	0.0369 (8)	0.0482 (9)	0.0697 (10)	0.0063 (7)	−0.0036 (7)	0.0084 (8)
C11	0.0350 (7)	0.0475 (8)	0.0548 (9)	−0.0042 (6)	0.0007 (6)	−0.0105 (7)
C12	0.0593 (11)	0.0459 (9)	0.0876 (13)	−0.0085 (8)	−0.0020 (10)	0.0046 (9)
C13	0.0622 (11)	0.0869 (14)	0.0731 (12)	−0.0139 (10)	0.0048 (10)	−0.0392 (11)

### 3-[2-(Propan-2-ylamino)ethyl]-1H-indol-4-ol (4) Geometric parameters (Å, º)

**Table:**

O1—C6	1.3618 (17)	C6—C7	1.4110 (18)
O1—H1	0.999 (9)	C7—C8	1.4415 (19)
N1—C1	1.363 (2)	C8—C9	1.498 (2)
N1—C2	1.367 (2)	C9—H9A	0.9700
N1—H1A	0.877 (9)	C9—H9B	0.9700
N2—C10	1.475 (2)	C9—C10	1.526 (2)
N2—C11	1.4774 (19)	C10—H10A	0.9700
N2—H2	0.897 (9)	C10—H10B	0.9700
C1—H1B	0.9300	C11—H11	0.9800
C1—C8	1.360 (2)	C11—C12	1.511 (2)
C2—C3	1.394 (2)	C11—C13	1.515 (2)
C2—C7	1.4131 (19)	C12—H12A	0.9600
C3—H3	0.9300	C12—H12B	0.9600
C3—C4	1.370 (2)	C12—H12C	0.9600
C4—H4	0.9300	C13—H13A	0.9600
C4—C5	1.399 (2)	C13—H13B	0.9600
C5—H5	0.9300	C13—H13C	0.9600
C5—C6	1.378 (2)		
			
C6—O1—H1	108.7 (12)	C8—C9—H9A	108.6
C1—N1—C2	108.74 (13)	C8—C9—H9B	108.6
C1—N1—H1A	123.5 (13)	C8—C9—C10	114.77 (13)
C2—N1—H1A	127.8 (14)	H9A—C9—H9B	107.6
C10—N2—C11	115.11 (12)	C10—C9—H9A	108.6
C10—N2—H2	109.6 (11)	C10—C9—H9B	108.6
C11—N2—H2	106.9 (11)	N2—C10—C9	112.54 (12)
N1—C1—H1B	124.3	N2—C10—H10A	109.1
C8—C1—N1	111.47 (14)	N2—C10—H10B	109.1
C8—C1—H1B	124.3	C9—C10—H10A	109.1
N1—C2—C3	129.74 (14)	C9—C10—H10B	109.1
N1—C2—C7	107.26 (13)	H10A—C10—H10B	107.8
C3—C2—C7	123.00 (13)	N2—C11—H11	108.8
C2—C3—H3	121.4	N2—C11—C12	108.75 (13)
C4—C3—C2	117.20 (14)	N2—C11—C13	109.86 (13)
C4—C3—H3	121.4	C12—C11—H11	108.8
C3—C4—H4	119.2	C12—C11—C13	111.73 (15)
C3—C4—C5	121.54 (15)	C13—C11—H11	108.8
C5—C4—H4	119.2	C11—C12—H12A	109.5
C4—C5—H5	119.3	C11—C12—H12B	109.5
C6—C5—C4	121.41 (14)	C11—C12—H12C	109.5
C6—C5—H5	119.3	H12A—C12—H12B	109.5
O1—C6—C5	119.70 (12)	H12A—C12—H12C	109.5
O1—C6—C7	121.38 (12)	H12B—C12—H12C	109.5
C5—C6—C7	118.92 (13)	C11—C13—H13A	109.5
C2—C7—C8	107.41 (12)	C11—C13—H13B	109.5
C6—C7—C2	117.91 (13)	C11—C13—H13C	109.5
C6—C7—C8	134.67 (13)	H13A—C13—H13B	109.5
C1—C8—C7	105.12 (14)	H13A—C13—H13C	109.5
C1—C8—C9	124.66 (14)	H13B—C13—H13C	109.5
C7—C8—C9	130.21 (13)		
			
O1—C6—C7—C2	178.65 (12)	C3—C2—C7—C8	−179.83 (13)
O1—C6—C7—C8	−0.4 (2)	C3—C4—C5—C6	0.5 (2)
N1—C1—C8—C7	0.54 (17)	C4—C5—C6—O1	−179.29 (13)
N1—C1—C8—C9	−179.63 (14)	C4—C5—C6—C7	0.1 (2)
N1—C2—C3—C4	179.42 (15)	C5—C6—C7—C2	−0.74 (19)
N1—C2—C7—C6	−178.91 (12)	C5—C6—C7—C8	−179.82 (14)
N1—C2—C7—C8	0.40 (15)	C6—C7—C8—C1	178.58 (15)
C1—N1—C2—C3	−179.82 (15)	C6—C7—C8—C9	−1.2 (3)
C1—N1—C2—C7	−0.08 (17)	C7—C2—C3—C4	−0.3 (2)
C1—C8—C9—C10	−104.54 (17)	C7—C8—C9—C10	75.2 (2)
C2—N1—C1—C8	−0.30 (19)	C8—C9—C10—N2	−72.62 (18)
C2—C3—C4—C5	−0.4 (2)	C10—N2—C11—C12	−166.98 (14)
C2—C7—C8—C1	−0.57 (15)	C10—N2—C11—C13	70.45 (18)
C2—C7—C8—C9	179.61 (14)	C11—N2—C10—C9	−115.33 (15)
C3—C2—C7—C6	0.9 (2)		

### 3-[2-(Propan-2-ylamino)ethyl]-1H-indol-4-ol (4) Hydrogen-bond geometry (Å, º)

**Table:**

*D*—H···*A*	*D*—H	H···*A*	*D*···*A*	*D*—H···*A*
N1—H1*A*···O1^i^	0.88 (1)	2.06 (1)	2.9217 (16)	167 (2)
O1—H1···N2	1.00 (1)	1.62 (1)	2.6217 (15)	176 (2)

Symmetry code: (i) −*x*+3/2, *y*+1/2, *z*.
